# Mechanism of Thiazide Diuretic Arterial Pressure Reduction: The Search Continues

**DOI:** 10.3389/fphar.2019.00815

**Published:** 2019-08-27

**Authors:** Robert M. Rapoport, Manoocher Soleimani

**Affiliations:** ^1^Department of Pharmacology and Systems Physiology, University of Cincinnati College of Medicine, Cincinnati, OH, United States; ^2^Research Service, Veterans Affairs Medical Center, Cincinnati, OH, United States; ^3^Department of Medicine, University of Cincinnati College of Medicine, Cincinnati, OH, United States

**Keywords:** hypertension, thiazide diuretics, diuresis, plasma volume, vasoconstriction, arterial blood pressure, renal, extrarenal

## Abstract

Thiazide diuretic (TZD)-mediated chronic reduction of arterial pressure is thought to occur through decreased total peripheral vascular resistance. Further, the decreased peripheral vascular resistance is accomplished through TZD activation of an extrarenal target, resulting in inhibition of vascular constriction. However, despite greater than five decades of investigation, little progress has been made into the identification of the TZD extrarenal target. Proposed mechanisms range from direct inhibition of constrictor and activation of relaxant signaling pathways in the vascular smooth muscle to indirect inhibition through decreased neurogenic and hormonal regulatory pathways. Surprisingly, particularly in view of this lack of progress, comprehensive reviews of the subject are absent. Moreover, even though it is well recognized that 1) several types of hypertension are insensitive to TZD reduction of arterial pressure and, further, TZD fail to reduce arterial pressure in normotensive subjects and animals, and 2) different mechanisms underlie acute and chronic TZD, findings derived from these models and parameters remain largely undifferentiated. This review 1) comprehensively describes findings associated with TZD reduction of arterial pressure; 2) differentiates between observations in TZD-sensitive and TZD-insensitive hypertension, normotensive subjects/animals, and acute and chronic effects of TZD; 3) critically evaluates proposed TZD extrarenal targets; 4) proposes guiding parameters for relevant investigations into extrarenal TZD target identification; and 5) proposes a working model for TZD chronic reduction of arterial pressure through vascular dilation.

## Introduction

Thiazide diuretics (TZD) are one of the most widely prescribed therapeutic agents for treatment of hypertension and are also used in the treatment of heart failure and stroke (Olde Engberink et al., 2015). However, the mechanism underlying chronic reduction of arterial pressure to TZD remains unclear despite investigations over greater than five decades. It is widely agreed that decreased total peripheral vascular resistance underlies TZD chronic pressure reduction (**Table 1a**). Moreover, an extrarenal target for TZD action has been proposed (**Table 1a**). However, there is little consensus on the extrarenal TZD target responsible for the chronic reduction of arterial pressure. Proposals range widely from indirect mechanisms, in which TZD inhibit regulatory sites upstream from the vasculature, to TZD direct inhibition of vascular constriction (**Table 1a**).

**Table 1:** Text citations^1^.

a. Tobian, 1967; Shah et al., 1978; Hughes, 2004; Ellison and Loffing, 2009; Duarte and Cooper-DeHoff, 2010; Tamargo et al., 2014; Shahin and Johnson, 2016; Pourafshar et al., 2018
b. Veterans Administration Cooperative Study on Antihypertensive Agents, 1962; de Carvalho et al., 1977; van Brummelen et al., 1980; Freis et al., 1988; Dudenbostel et al., 2017; Pathare et al., 2017
c. Preziosi et al., 1959; Friedman et al., 1960; Preziosi et al., 1961; Daniel, 1962; Tobian and Coffee, 1964; Stanton and White, 1965; Lockett and Nicholas, 1968; Aoki and Brody, 1969; Nicholas, 1971; Finch et al., 1977; Ballew and Fink, 2001; Li and Wang, 2007; Sládková et al., 2007; Jessup et al., 2008; Ashek et al., 2012; Calhoun, 2013; Yu et al., 2015; Araos et al., 2016; Siddiqui et al., 2016
d. Freis et al., 1958; Wilkins et al., 1958; Dollery et al., 1959; Freis, 1959; Preziosi et al., 1959; Freis et al., 1960; Friedman et al., 1960; Fuchs et al., 1960; Hollander et al., 1960; Mendlowitz et al., 1960; Preziosi et al., 1961; Lockett and Nicholas, 1968; Aoki and Brody, 1969; Neuvonen, 1971; Jandhyala et al., 1972; Shah et al., 1978; Ballew and Fink, 2001; Li and Wang, 2007; Jessup et al., 2008
e. Hughes, 2004; Ellison and Loffing, 2009; Duarte and Cooper-DeHoff, 2010; Tamargo et al., 2014; Shahin and Johnson, 2016; Pourafshar et al., 2018
f. Leviel et al., 2010; Eladari and Chambrey, 2011; Eladari and Hübner, 2011; Sinning et al., 2017).
g. Freis et al., 1958; Dustan et al., 1959; Dollery et al., 1959; Freis, 1959; Friedman et al., 1960; Frohlich et al., 1960; Fuchs et al., 1960; Hollander et al., 1960; Shah et al., 1978; Ballew and Fink, 2001; Jessup et al., 2008
h. Freis et al., 1958; Wilson and Freis, 1959; Fuchs et al., 1960; Hollander et al., 1960; Talso and Carballo, 1960; Winer, 1961
i. Wilson and Freis, 1959; Conway and Lauwers, 1960; Lauwers and Conway, 1960; Gifford et al., 1961; Leth, 1970, Lund-Johansen, 1970; Shah et al., 1978; van Brummelen et al., 1980
j. Freis et al., 1958; Wilkins et al., 1958; Dollery et al., 1959; Freis, 1959; Preziosi et al., 1959; Freis et al., 1960; Friedman et al., 1960; Fuchs et al., 1960; Hollander et al., 1960; Mendlowitz et al., 1960; Preziosi et al., 1961; Joubert et al., 1968; Lockett and Nicholas, 1968; Aoki and Brody, 1969; Neuvonen, 1971; Jandhyala et al., 1972; Claus-Walker et al., 1977; Shah et al., 1978; Ballew and Fink, 2001; Li and Wang, 2007
k. Colussi et al., 1997; Colussi et al., 2007; Peng et al., 2016; Peng et al., 2017; Peng et al., 2018
l. Calò et al., 1998; Calò et al., 1999; Simon et al., 1996; Calò, 2006; Riveira-Munoz et al., 2007a; Riveira-Munoz et al., 2007b; Wang et al., 2015b; Peng et al., 2017
m. Bourgoignie et al., 1968; Tarazi et al., 1970; van Brummelen et al., 1980; Lijnen et al., 1981; Xiao et al., 2013
n. Conway and Lauwers, 1960; Greene et al., 1961 ; Villarreal et al., 1962; Lund-Johansen, 1970; de Carvalho et al., 1977; Shah et al., 1978; Lake et al., 1979; van Brummelen et al., 1980
o. Tapia et al., 1957; Freis et al., 1958; Dollery et al., 1959; Dustan et al., 1959; Gifford et al., 1961; Winer, 1961; Greene et al., 1964
p. Grossman et al., 1994; Dollery et al., 1959; Holland et al., 1983; Borghi and Omboni, 2014; Yu et al., 2015
q. Meier et al., 1957; Beavers and Blackmore, 1958; Preziosi et al., 1959; Crosley et al., 1960; Freis et al., 1960; Hollander et al., 1960; Overbeck and Haddy, 1960; Preziosi et al., 1961; Gillenwater et al., 1962; Greene et al., 1963; Greene et al., 1964; Silah et al., 1965; Lockett and Nicholas, 1968; Nicholas, 1971; Pickkers et al., 1998).
r. Daniel and Nash, 1965; Nicholas, 1970; Shah et al., 1978; Mironneau et al., 1981; Calder et al., 1992; Calder et al., 1993; Calder et al., 1994; Pickkers and Hughes, 1995; Abrahams et al., 1996; Abrahams et al., 1998; Pickkers et al., 1999; Colas et al., 2000a; Colas et al., 2000b; Colas et al., 2000c; Colas et al., 2001; Zhu et al., 2005; Sládková et al., 2007; Afsar et al., 2016; Shahin and Johnson, 2016; Alshahrani et al., 2017a; Rapoport et al., 2019
s. Shah et al., 1978; Mironneau et al., 1981; Calder et al., 1992; Calder et al., 1993; Calder et al., 1994; Pickkers and Hughes, 1995; Abrahams et al., 1996; Abrahams et al., 1998; Pickkers et al., 1999; Colas et al., 2000a; Colas et al., 2000b; Colas et al., 2000c; Colas et al., 2001; Zhu et al., 2005; Sládková et al., 2007; Afsar et al., 2016; Shahin and Johnson, 2016
t. Mendlowitz et al., 1960; McCubbin and Page, 1963; Baum and Shropshire, 1967; Aoki and Brody, 1969; Lake et al., 1979

^1^Statements in text associated with greater than or equal to 5 citations are grouped and indicated as “a”, “b”,...“t”. Letters are referred to in text as (**Table 1a**), (**Table 1b**),...(**Table 1t**), respectively. Statements in the text associated with less than 5 citations are not grouped and, therefore, are not included as lettered entries and remain in the text.

**Figure 1:**
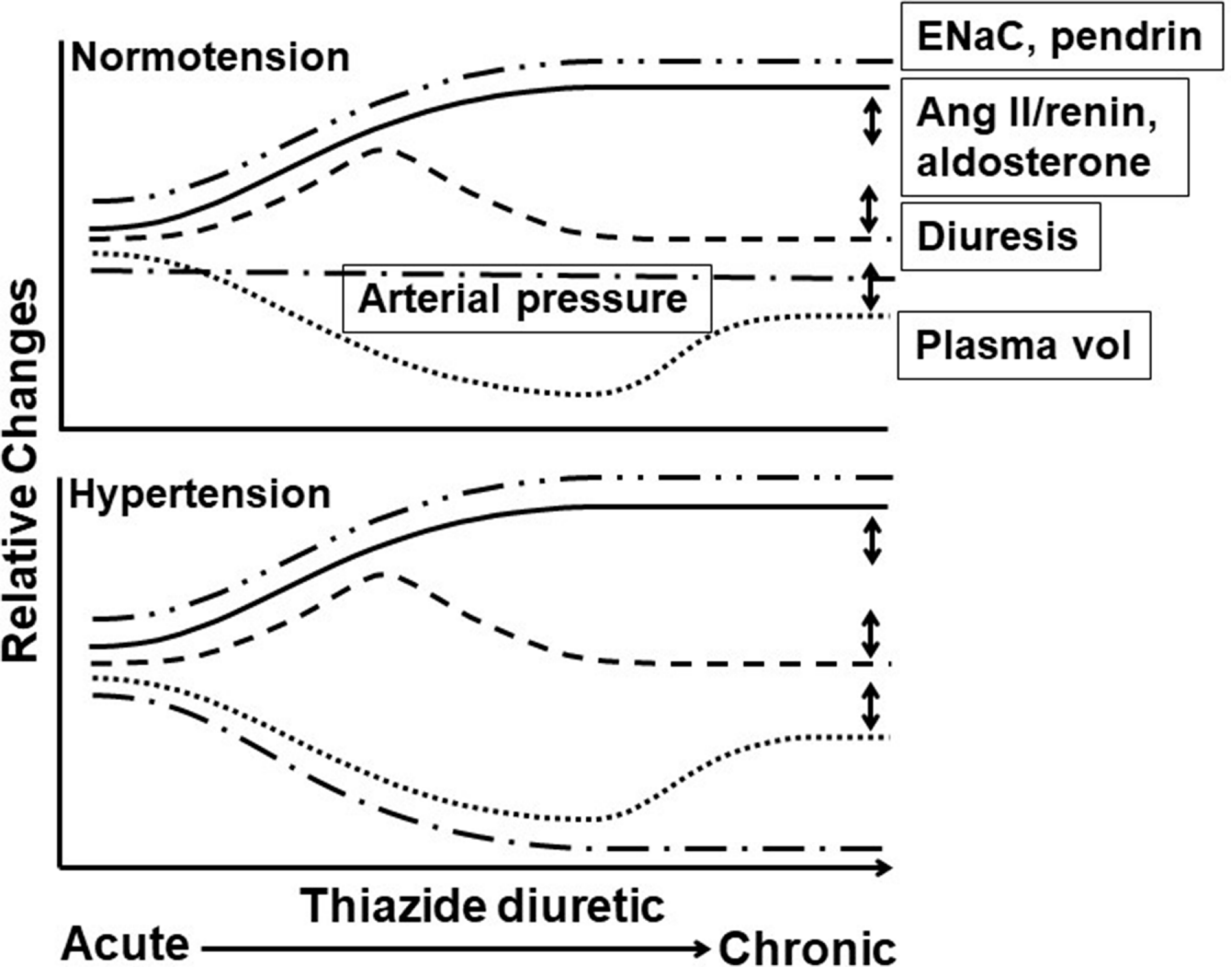
Time course of thiazide diuretic effects on arterial pressure, plasma volume, diuresis, and levels of angiotensin II/renin, ENaC, and pendrin in hypertension in responders and normotension. Acute through chronic time course of thiazide diuretic (TZD) effects in normotension (top panel) and hypertension (bottom panel) on relative changes in arterial pressure (**^__.__^**), plasma volume (**^…^**), renin activity/angiotenisn II and aldosterone plasma levels (**^_____^**), diuresis (----), and epithelial sodium channel (ENaC) and pendrin expression (**^__..^**)**^.^** Features of the time course include **(1)** plasma volume: decreased with acute TZD and partial return to pre-TZD level with chronic TZD, with similar magnitude and time course in normotension and hypertension; **(2)** arterial pressure: reduced with acute and chronic TZD in hypertension and unaffected in normotension despite similar changes in plasma volume and diuresis; **(3)** renin activity/angiotensin II and aldosterone plasma levels: increased with acute and chronic TZD. Angiotensin II/renin and aldosterone levels in normotension with TZD have not been measured (to our knowledge) and, therefore, are speculated based upon the decreased plasma volume; and **(4)** ENaC and pendrin expression: increased with acute and chronic TZD, with similar magnitude and time course in normotension and hypertension. Increased ENaC and pendrin expression likely compensate for the Double-headed arrows indicate variable effects. TZD-induced diuresis mediated through NCC inhibition. Only single time points for ENaC and pendrin have been reported, and thus, the time course is speculative. Double-headed arrows indicate variable effects. See text for additional details.

Likely contributing to the wide range of proposed TZD targets for chronic reduction of arterial pressure and, furthermore, largely overlooked, are the use of the following:

1) Hypertensive models that are sensitive and insensitive to TZD arterial pressure reduction. In this regard, TZD reduce arterial pressure in some hypertensive patients, while TZD lack efficacy in others, i.e., “responders” and “nonresponders,” respectively (**Table 1b**). Indeed, as great as 20% of hypertensive patients are resistant to TZD arterial pressure reduction (Dudenbostel et al., 2017). Similarly, models of hypertension can be sensitive (1 kidney/DOCA-salt, 1 kidney/DOCA, DOCA-salt, angiotensin II-salt, spontaneous hypertensive rats, dietary salt in rats, NO synthase inhibitor, *cyp1a1-Ren2* hypertensive rats, capsaicin-high salt, and low renin hypertension) or insensitive (renal, e.g., following renal artery constriction; neurogenic/sympathetic nervous system, e.g., following aortic depressor plus sinus nerve ligation and vagi-aortic nerve ligation, increased dietary salt in mice, angiotensin II, and hyperaldosteronism) to TZD arterial pressure reduction (**Table 1c**). Indeed, overall predictors of greater responsiveness to TZD include lower levels of plasma renin and urine aldosterone (Chapman et al., 2002). Clearly, the efficacy of TZD and other antihypertensive agents to reduce arterial pressure depends upon the target sites in the different types of hypertension (Gong et al., 2012).
2) Normotensive subjects and animals. TZD fail to reduce arterial pressure in normotensive subjects/animals (**Table 1d**; **Figure 1**).
3) Acute TZD challenge. Contrasting mechanisms underlie TZD acute and chronic reduction of arterial pressure, with the former renal mediated, caused by diuresis and accompanying decreased plasma volume, and the latter in which arterial pressure reduction and plasma volume depletion are dissociated (**Table 1a**; **Figure 1**).
4) Supra-therapeutic dose/concentration of TZD. At therapeutic dose, TZD selectively inhibit the Na^+^/Cl^−^ cotransporter (NCC; *SLC12A3*; Sinning et al., 2017). NCC, which is expressed on the apical membrane of distal convoluted tubule cells, is the renal target responsible for TZD diuresis (**Table 1e**). However, at supra-therapeutic dose, TZD also inhibit the a) Na^+^-dependent chloride–bicarbonate exchanger (NDCBE; *SLC4A8*; **Table 1f**). NDCBE, which is located on the basolateral membrane of medullary collecting duct cells, regulates cell pH (Xu et al., 2018) and b) carbonic anhydrase isozymes (Swenson, 2014). Carbonic anhydrase isoenzymes are located in the renal proximal tubule and intercalated cells, where they mediate bicarbonate transport, as well as extrarenally (Swenson, 2014). Also, a number of *in vivo* studies on the effects of TZD on arterial blood pressure and a large majority of *in vitro* studies of the effects of TZD on the vasculature generally utilized supra-therapeutic TZD concentrations [(Na+/Cl^−^Cotransporter (NCC; SLC1283), *In Vivo* TZD on Vascular Contractility Determined In Vitro].

This review 1) comprehensively describes findings related to TZD reduction of arterial pressure; 2) differentiates between observations derived from TZD-sensitive and TZD-insensitive hypertensive models, normotensive subjects/animals, acute and chronic TZD, and TZD dose/concentration; 3) proposes guiding parameters for clinically relevant, extrarenal TZD target identification; 4) critically evaluates proposed TZD extrarenal targets; and 5) proposes a working model for TZD chronic reduction of arterial pressure through dilation of the vasculature.

### (1) Dose/Concentration of TZD

The use of supra-therapeutic doses/concentrations in *in vivo* and *ex vivo* studies undoubtedly contributes to the lack of clarity regarding the mechanism of TZD chronic reduction of arterial pressure. In order to clarify this potential impact, it is important to initially establish the values which constitute supra-therapeutic TZD doses/concentrations.

#### Therapeutic TZD Doses/Concentrations

TZD doses exceeded those required for maximal efficacy in early years of TZD use (Tamargo et al., 2014). Subsequently, TZD doses were substantially decreased in order to lower the incidence of side effects, with the overwhelming majority of TZD doses established at less than *p.o*. 50 mg/day (approximately 0.65 mg/kg/day; Musini et al., 2014; Tamargo et al., 2014; Vongpatanasin, 2014).

Bioavailability of TZD varies from 65% to 95% (Tamargo et al., 2014). Treatment of hypertension with *p.o.* hydrochlorothiazide 7.5–25 mg/day (approximately 0.1–0.3 mg/kg/day) resulted in a median plasma concentration of 0.26 µM (Sigaroudi et al., 2018). Consistent with the plasma hydrochlorothiazide level derived at these doses (Sigaroudi et al., 2018), treatment of hypertension and congestive heart failure with *p.o.* 75 mg/day (approximately 1 mg/kg/day) and 50 mg/day (approximately 0.7 mg/kg/day), respectively, yielded peak blood levels of approximately 1–3 µM (Beermann and Groschinsky‐Grind, 1977; Beermann and Groschinsky‐Grind, 1978; Beermann and Groschinsky‐Grind, 1979).

### (2) Diuresis and Plasma Volume

Fundamental to TZD reduction of arterial pressure and, thus, elucidation of the TZD extrarenal target, is increased diuresis and resultant decreased plasma volume. Characteristics of TZD-induced diuresis and decreased plasma volume include differentiation into acute and chronic phases and associated underlying mechanisms.

#### Responders and TZD-Sensitive Hypertension Models

Acute TZD reduction of arterial pressure correlates with diuresis and the associated decreased plasma volume in hypertensive humans and animals (**Table 1g**; **Figure 1**). Exceptions to this correlation are TZD pressure reduction prior to decreased plasma volume (Gifford et al., 1961; Greene et al., 1961; Lockett and Nicholas, 1968).

Plasma volume repletion reversed acute TZD reduction of arterial pressure, consistent with the correlation between acute arterial pressure reduction and diuresis (**Figure 1**). Specifically, after TZD treatment for 6 days, dextran infusion increased arterial pressure as well as the cardiac index and reduced total peripheral vascular resistance in humans (Dustan et al., 1959). However, after TZD treatment for 7 days, in which the plasma volume decrease was maintained at the initial reduced level, plasma and saline infusion did not alter arterial pressure (Hollander et al., 1960). Also, in barbiturate-anesthetized 1 kidney/DOCA hypertensive rats challenged with TZD for 5–8 days, hypotonic saline and dextrose infusion did not reverse the reduced arterial pressure, although hypertonic saline infusion increased systolic pressure, albeit not diastolic pressure (Daniel, 1962).

In contrast to acute TZD, chronic TZD reduction of arterial pressure appears dissociated from diuresis and decreased plasma volume (**Figure 1**). With respect to diuresis, TZD chronic reduction in arterial pressure is maintained while diuresis is minimized in hypertensive humans (**Table 1h**). TZD also did not alter serum Na^+^ (**Table 1h**). Additionally, water and Na^+^ balance returned to normal during TZD challenge of hypertensive high Na^+^ diet/angiotensin II rats and spontaneously hypertensive rats, while reduced arterial pressure was maintained (Ballew and Fink, 2001; Jessup et al., 2008). Also consistent with the dissociation between diuresis and chronic TZD reduction of arterial pressure is the lesser diuretic efficacy of TZD than inhibitors of the luminal Na–K–Cl cotransporter (NKCC2) located in the thick ascending limb of Henle (loop diuretics), i.e., the NCC and NKCC2 regulate approximately 7% and 25% of Na^+^ absorption, respectively (Glover and O’Shaughnessy, 2013), while the antihypertensive efficacy of TZD was greater than that of NKCC2 inhibitors (Anderson et al., 1971; Araoye et al., 1978; Holland et al., 1979). Contributing to the minimal diuresis is increased expression of epithelial Na^+^ channel (ENaC) and Na^+^ anion exchanger, pendrin (Na et al., 2003; Alshahrani and Soleimani, 2017; **Figure 1**).

With respect to plasma volume, TZD-decreased plasma volume apparently returned to pre-TZD level despite continuous TZD for 1–12 months in hypertensive humans (**Table 1i**; **Figure 1**). Whether, in fact, plasma volume completely or only partially returned to pre-TZD level is unclear because a number of these studies demonstrated a tendency for reduced plasma volume. Moreover, the magnitude of plasma volume return to pre-TZD level may be overestimated by increased hemoglobin (Hilden et al., 1968; Leth, 1970). Along these lines, the TZD lowered plasma volume only partially returned at 2–3 months of TZD treatment (Winer, 1961; Hansen, 1968), and as determined at a single time point during TZD treatment, plasma volume was decreased at 12 and 24 months of TZD (Lund-Johansen, 1970; Tarazi et al., 1970; de Carvalho et al., 1977).

Also consistent with the only partial return of plasma volume with chronic TZD is the return of TZD-reduced arterial pressure following plasma volume expansion with dextran plus saline or glucose infusion at 2 weeks to 8 months of TZD treatment in hypertensive humans (Wilson and Freis, 1959). In partial agreement, following 2 weeks to 9 months of TZD treatment, dextran plus glucose infusion resulted in partial return of systolic pressure while the decreased diastolic pressure remained (Winer, 1961).

Additionally, plasma volume repletion with elevated dietary Na^+^ reversed TZD chronic reduction of arterial pressure in hypertensive humans (Wilkins et al., 1958). At 6 days post single TZD injection (*i.v*. 1 g of chlorothiazide), increased dietary Na^+^ of 140 mEq/day (1.8 g/day) from previously lowered 9 mEq/day (0.2 g/day), completely reversed the sustained reduction of arterial pressure to pre-TZD level (Wilkins et al., 1958). In partial agreement, greater amounts of dietary Na^+^ salt, i.e., 6–12 g/day (2.4–4.8 g/day Na^+^) only partially reversed TZD-mediated arterial pressure reduction, and complete reversal required 20 g/day (8 g/day Na^+^; Freis et al., 1958; Wilkins et al., 1958; Freis, 1959; Winer, 1961). Also, following withdrawal of TZD, plasma volume increases (Wilson and Freis, 1959; Tarazi et al., 1970).

Apparently inconsistent with the correlation between elevated Na^+^ and reversal of TZD-reduced arterial pressure, TZD reduction of arterial pressure remained despite prevention of Na^+^ loss by mineralocorticoid co-administration (Hollander et al., 1960). However, it is likely that TZD-decreased plasma volume was actually maintained because weight loss, an indirect measure of plasma volume, also remained following mineralocorticoid co-administration (Hollander et al., 1960).

Decreased Na^+^ excretion as the result of Na^+^-restricted diet (urinary excretion from 3.75 g Na^+^/day to 2 g Na^+^/day) which was associated with reduced arterial pressure and increased renin activity and aldosterone plasma level, also failed to prevent further arterial pressure reduction by TZD in the absence or presence of angiotensin receptor antagonists in humans (Priddle et al., 1970; Vogt et al., 2008; Wang et al., 2015b). The TZD arterial pressure reduction was still associated with plasma volume contraction because Na^+^-restricted diet did not prevent TZD-increased Na^+^ excretion (urine volume not reported) and futher increased plasma renin activity and aldosterone levels (Priddle et al., 1970; Vogt et al., 2008).

#### Summary and Conclusions: Responders and TZD-Sensitive Hypertension Models

**Figure 2:**
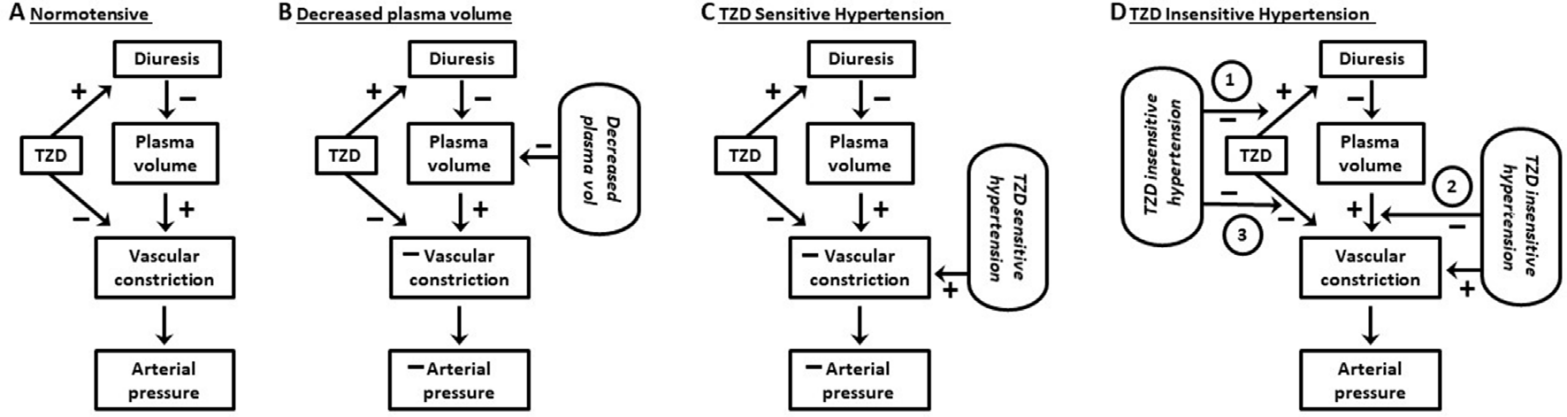
*Working model of effects of thiazide diuretics on vascular constriction and arterial pressure.* **(A)** Normotensive subjects: thiazide diuretics (TZD) induce diuresis but fail to reduce arterial pressure. The lack of pressure reduction results from a balance between vasoconstriction due to (1) signaling pathways activated in response to TZD decrease in plasma volume and (2) TZD direct inhibition of the vasoconstriction. **(B)** Normotensive subjects and decreased plasma Na^+^: vasoconstriction due to activation of compensatory regulatory pathways in response to decreased plasma Na^+^ by Na^+^-restricted diet plus TZD-induced diuresis is unable to mitigate the reduction in arterial pressure caused by TZD direct inhibition of the constriction. **(C)** TZD-sensitive hypertension: TZD reduce arterial pressure due to TZD direct inhibition of vasoconstriction overcoming constriction due to both plasma volume contraction (in response to decreased plasma volume) and hypertension. **(D)** TZD-insensitive hypertension: TZD fail to reduce arterial pressure due to inhibition of diuresis, plasma volume contraction, and/or prevention of TZD inhibition of vascular constriction (circled 1, 2, and 3, respectively). See text for additional details.

Among the findings in support of an extrarenal, vascular mechanism mediating TZD chronic reduction of arterial pressure in hypertensive humans and animals is the maintained pressure reduction despite return of plasma volume towards pre-TZD levels (**Figure 1**). Overall, however, it appears that a remaining component of TZD acute decreased plasma volume is required for chronic TZD reduction in arterial pressure (**Figures 1**, **2**). Thus, TZD chronic reduction of arterial pressure is dependent upon decreased plasma volume both as a trigger for the subsequent maintained arterial pressure reduction and for its ability to sustain the reduction (**Figure 2**).

#### Normotensive Humans and Animals

Acute and chronic TZD failed to reduce arterial pressure in normotensive humans and animals despite the diuresis and decreased plasma volume (**Table 1j**; **Figure 1**). A supra-therapeutic TZD dose was required to reduce arterial pressure in dogs (Preziosi et al., 1959). Lack of TZD reduction of arterial pressure was not due to decreased diuresis because TZD caused similar magnitudes of changes in Na^+^ and K^+^ per body weight, as well as extracellular fluid volume and serum osmolarity, in normotensive subjects and hypertensive patients (Freis et al., 1960; Hollander et al., 1960; **Figure 1**). Diuresis due to NKCC2 inhibitors was also greater than diuresis due to TZD in normotensive subjects, while NKCC2 inhibitors still failed to reduce arterial pressure (Joubert et al., 1968).

In apparent contrast to the lack of TZD reduction of arterial pressure in normotensive humans and animals (**Table 1j**; **Figure 1**), in hypertensive humans. TZD and Na^+^-restricted diet elicited a steady reduction of arterial pressure (Priddle et al., 1970). Na^+^-restricted diet did not prevent TZD diuresis and natriuresis in rats (Hofmann and Sagartz, 1970) and, thus, were presumably also not prevented in humans.

Although TZD did not cause diuresis in normotensive mice, the absence of diuresis may actually reflect the relatively delayed assessment, i.e., day 3–4 post initial TZD (Alshahrani and Soleimani, 2017). This suggestion is supported by the return to baseline within 24 and 72 h of increased diuresis due to *p.o*. TZD *q.d*. (Fuchs et al., 1960; Claus-Walker et al., 1977) and 48 h *p.o*. TZD *b.i.d*. (Wilson and Freis, 1959). Also, ENaC and pendrin expression, which presumably compensates for TZD diuresis through increased Na^+^ reabsorption, increased on day 3 post initial TZD treatment (earliest time point assayed; Alshahrani and Soleimani, 2017; **Figure 1**). Along these lines, diuresis increased 2 h post a single *i.v*. injection of TZD in rats and ENaC activity remained unchanged (Frindt et al., 2017).

#### Summary and Conclusions: Normotensive Humans and Animals

TZD failed to reduce arterial pressure in normotensive humans and animals despite similar magnitudes of diuresis and plasma volume decrease as in hypertensive humans and animals (**Figures 1**, **2**). The lack of TZD reduction of arterial pressure suggests a balance between compensatory contraction of the plasma volume and TZD mitigation of the contracted volume through inhibition of vasoconstriction (**Figure 2**). However, this balance is disrupted by further plasma volume contraction, which can occur, e.g., with increased Na^+^ loss, resulting in TZD reduction of arterial pressure (**Figure 2**).

#### Responders versus Nonresponders

TZD-mediated increased diuresis and plasma volume reduction, and body weight loss were greater in responders than nonresponders (de Carvalho et al., 1977; Freis et al., 1988; **Table 2**). Also, TZD failed to decrease plasma volume in a study limited to nonresponders (Svendsen et al., 1983). The greater contracted plasma volume of responders was suggested to underlie increased TZD reduction of total peripheral vascular resistance (de Carvalho et al., 1977). Consistent with the lesser magnitude of plasma volume contraction in nonresponders is the lack of angiotensin II and aldosterone plasma level elevation (Svendsen et al., 1983; Constrictor Pathways *In Vivo*, Endogenous Activation; **Table 2**). The lesser decrease in plasma volume in nonresponders than responders was not associated with lesser decreases in serum K^+^ (de Carvalho et al., 1977; Svendsen et al., 1983).

**Table 2:** Effects of Thiazide Diuretics in Responders and Nonresponders on Physiological and Biochemical Parameters in Hypertension.

Effect	Responder vs Non-responder^1^	Reference
NCC ^2^ expression/phosphorylation	>	Pathare et al., 2017
Diuresis	>	Freis et al., 1988
Plasma volume decrease	>	de Carvalho et al., 1977
	>	Freis et al., 1988
	≡^3^	van Brummelen et al., 1980
Weight loss	≡	van Brummelen et al., 1980
	>	Freis et al., 1988
Renin/angiotensin II	≡^3^	van Brummelen et al., 1980
	≡^3,4^	Freis et al., 1988
Aldosterone	<	van Brummelen et al., 1980
Cardiac/sympathetic	≡	de Carvalho et al., 1977
	>	van Brummelen et al., 1980

^1^Greater, less, equivalent: >, <, ≡; ^2^NCC = Na^+^/Cl^-^ cotransporter; ^3^Tended to be less; ^4^Extrapolation based on equivalent TZD dose.

In contrast, there was a tendency for increased plasma volume contraction in chronic TZD-treated nonresponders than responders (van Brummelen et al., 1980; **Table 2**). It was suggested that the tendency for greater plasma volume contraction in nonresponders, which was associated with a tendency for greater elevated plasma levels of angiotensin II (Constrictor Pathways *In Vivo*, Endogenous Activation; **Table 2**), resulted in increased peripheral vascular resistance (van Brummelen et al., 1980) and, thus, diminished TZD-decreased arterial pressure. On the other hand, body weight was not different in responders and nonresponders (van Brummelen et al., 1980; **Table 1**).

#### Summary and Conclusions: Responders versus Nonresponders

The dependency of TZD reduction of arterial pressure upon relaxation of vasoconstriction suggests that the inability of TZD to reduce arterial pressure in nonresponders may reflect the lack of compensatory vasoconstriction (**Table 2**; **Figure 2**). That is, the inability of TZD to elicit diuresis results in lack of decreased plasma volume and, therefore, absence of compensatory vasoconstriction (**Table 2**; **Figure 2**). This conclusion is supported by the lack of TZD arterial pressure reduction in normotensive humans and animals despite increased diuresis (above). Additionally, lack of diuresis in some types of hypertension associated with nonresponders could result from prevention of diuresis (**Table 2**; **Figure 2**).

Alternatively, TZD may induce diuresis in TZD-insensitive hypertension with different etiologies. Thus, the absence of arterial pressure reduction could result from inhibitory effects post plasma volume depletion (**Figure 2**). Additional studies of the effects of TZD in nonresponders with different hypertensive etiologies are warranted, including the mechanism underlying TZD reduction of arterial pressure in nonresponders with co-administration of other antihypertensive agents (Sander and Giles, 2011; Tamargo et al., 2014).

### (3) Na^+^/Cl^−^ Cotransporter (NCC; *SLC12A3*)

Insight into the mechanism underlying TZD chronic reduction of arterial pressure may be derived 1) through the effects of TZD on the activity of NCC, the renal target for TZD diuresis (**Table 1e**), and the relationship between NCC activity and arterial pressure in responders and nonresponders; 2) in NCC knockout and NCC/pendrin double-knockout mice (Schultheis et al., 1998; Loffing et al., 2004; Soleimani et al., 2012; Alshahrani et al., 2017a); and 3) in humans with dysfunctional NCC, i.e., Gitelman’s syndrome (**Table 1l**);.

#### NCC Activity Determinations

NCC activity determinations include increased NCC expression and phosphorylation, with the latter an indicator of NCC trafficking (Glover and O’Shaughnessy, 2013; Pathare et al., 2017). These two measurements may be unrelated because phosphorylation can be dissociated from increased NCC expression (Frindt et al., 2017). TZD binding to NCC, determined by high-affinity binding with ^3^H-metolazone (Beaumont et al., 1988), has also been used as an indirect measure of NCC expression and, thus, activity (Chen et al., 1990; Morsing et al., 1991). However, even though TZD increased NCC binding, transport was reduced (Morsing et al., 1991). Additional potential complications with binding studies include the inability to account for NCC isoforms along with selective isoform phosphorylation (Tutakhel et al., 2016; Pathare et al., 2017), as well as the influence of Na^+^ and Cl^−^ concentrations (Beaumont et al., 1988; Tran et al., 1990).

#### Responders

In responders, chronic TZD increased NCC expression and NCC phosphorylation, as assayed in urinary vesicular exosomes (Pathare et al., 2017).

#### Normotensive Animals

Acute and chronic TZD increased NCC expression and phosphorylation in whole kidney and, in some studies, distal convoluted tubules from normotensive rats and mice (Northern and Western blot, *in situ* hybridization, and immunohistochemistry; Loffing et al., 1996; Na et al., 2003; Nijenhuis et al., 2005; Frindt et al., 2017). TZD also increased ^3^H-metolazone high-affinity binding to NCC in kidney membrane fractions from normotensive rats (Chen et al., 1990; Morsing et al., 1991).

Enhanced delivery of Na^+^ to the distal collecting duct likely underlies the increased NCC expression (Pathare et al., 2017) because furosemide, a diuretic that acts through inhibition of NKCC2, also increased NCC expression and binding in rat kidney (Chen et al., 1990; Na et al., 2003). Also, angiotensin receptor antagonists failed to increase NCC expression and phosphorylation even though arterial pressure was reduced to a similar magnitude as TZD (Pathare et al., 2017).

#### Responders versus Nonresponders

TZD-induced NCC expression and phosphorylation were greater in responders than nonresponders, as determined in urinary vesicular exosomes (Pathare et al., 2017; **Table 1**).

#### Summary and Conclusions: Responders, Normotensive Animals, and Responders versus Nonresponders

Increased NCC expression and phosphorylation due to TZD are consistent with the greater amount of diuresis in responders than nonresponders (Freis et al., 1988; **Table 2**; **Figure 2**; Diuresis and Plasma Volume). In normotensive animals, there is also a positive relationship between TZD-mediated NCC expression/phosphorylation and diuresis (Diuresis and Plasma Volume). NCC activation is restricted to agents that increase Na^+^ delivery to the distal collecting duct.

#### NCC Knockout and NCC/Pendrin Double-Knockout Mice

NCC knockout mice demonstrated changes similar to those observed in Gitelman’s syndrome, in which NCC is dysfunctional (below), including hypomagnesemia, hypocalciuria, and increased renin expression (determined in the kidney; Schultheis et al., 1998; Loffing et al., 2004; Alshahrani et al., 2017a). It should be noted, however, that TZD increased NCC expression and that NCC expression is decreased in Gitelman’s syndrome (Joo et al., 2007; Isobe et al., 2013; Yang et al., 2013; Frindt et al., 2017). This difference in NCC expression with TZD and Gitelman’s syndrome may reflect different mechanisms whereby TZD inhibit NCC activity (Payne, 2012) and of NCC dysfunction in Gitelman’s syndrome (Riveira-Munoz et al., 2007a, Riveira-Munoz et al., 2007b; Wang et al., 2015a).

Plasma bicarbonate levels were also elevated in NCC knockout mice, indicative of underlying, compensated alkalosis (Loffing et al., 2004). Additionally, aldosterone plasma levels were elevated (Loffing et al., 2004), although elevated aldosterone plasma levels were not consistently observed (Schultheis et al., 1998).

Basal arterial pressure: Basal arterial pressure was not significantly decreased in NCC knockout mice, although there was a tendency for decreased arterial pressure (Schultheis et al., 1998). The absence of hypotension in NCC knockout mice was attributed to compensation of mildly decreased intravascular volume (Schultheis et al., 1998; Loffing et al., 2004; Alshahrani et al., 2017a). Lack of hypotension in NCC knockout mice (Schultheis et al., 1998; Loffing et al., 2004; Alshahrani et al., 2017a), but hypotension in 50% of Gitelman’s syndrome patients (Cruz et al., 2001; below), may reflect differences in Na^+^ handling in mice and humans. This suggestion is supported by the arterial pressure reduction with Na^+^-restricted diet still containing 77.5% of normal in humans (Priddle et al., 1970), but the absence of arterial pressure reduction by more severe Na^+^-restricted diet of 0.01% and 0.1% in wild-type mice (Schultheis et al., 1998; Alshahrani et al., 2017a).

In contrast, arterial pressure was lower in NCC knockout mice with Na^+^-restricted diet (0.01% or 0.1%; Schultheis et al., 1998; Alshahrani et al., 2017a). Thus, it appears that compensatory vasoconstrictor pathways were unable to fully normalize the lower arterial pressure in NCC knockout mice subjected to Na^+^-restricted diet despite further increased renin expression (Schultheis et al., 1998; Alshahrani et al., 2017a). Similarly, NCC/pendrin double-knockout mice, which demonstrated severe diuresis and natriuresis along with even greater renin expression, was associated with further reduced arterial pressure (Soleimani et al., 2012).

TZD on arterial pressure: Hydrochlorothiazide failed to reduce arterial pressure in NCC knockout mice, despite *s.c.* administration at the supra-therapeutic dose of 20 mg/kg, i.e.*,* approximately 30-fold greater than the therapeutic dose (Alshahrani et al., 2017a; Dose/Concentration of TZD). In fact, 20 mg/kg of hydrochlorothiazide would result in a plasma concentration of approximately 50 µM (Dose/Concentration of TZD). Lack of hydrochlorothiazide reduced arterial pressure in NCC knockout mice may reflect conditions similar to those associated with normotensive subjects and animals (**Figure 2**). That is, vasoconstriction available for TZD relaxation is minimal due to the relatively small magnitude of decreased intravascular volume and, thus, minimal compensatory vasoconstriction (**Figure 2**).

In contrast, hydrochlorothiazide (20 mg/kg) reduced arterial pressure in NCC knockout mice subjected to Na^+^-restricted diet (Alshahrani et al., 2017a). Moreover, in NCC/pendrin double-knockout mice, hydrochlorothiazide elicited an even greater reduction in arterial pressure than in NCC knockout mice subjected to Na^+^-restricted diet (Alshahrani et al., 2017a). Thus, the magnitude of hydrochlorothiazide arterial pressure reduction correlated with greater underlying vasoconstriction as the result of increased intravascular volume depletion (Soleimani et al., 2012; Alshahrani et al., 2017a; **Figure 2**). Consistent with this suggestion is that prevention of intravascular volume depletion with increased dietary Na^+^ from 1% to 7% abolished both the lowered basal arterial pressure and TZD reduction of arterial pressure in NCC/pendrin double-knockout mice (Alshahrani et al., 2017a).

It was suggested that TZD reduction of arterial pressure in the absence of NCC provided evidence for an extrarenal TZD target (Alshahrani et al., 2017a). Further, hydrochlorothiazide arterial pressure reduction does not appear mediated by decreased plasma volume, due to decreased plasma Na^+^ (Alshahrani et al., 2017a). This conclusion is based upon the finding that urine production was actually decreased in NCC/pendrin double-knockout mice (Alshahrani et al., 2017a). The decreased urine production was attributed to lowered kidney perfusion with reduced arterial pressure (Alshahrani et al., 2017a). Also contributing to the decreased urine production was lowered water intake (Alshahrani et al., 2017a). Inexplicably, food intake was greatly decreased by hydrochlorothiazide (Alshahrani et al., 2017a).

Measurements of plasma Na^+^ would further test whether decreased plasma volume through lowered plasma Na^+^ was involved in hydrochlorothiazide reduction of arterial pressure in NCC knockout mice subjected to Na^+^-restricted diet and NCC/pendrin double-knockout mice (Alshahrani et al., 2017a). In this regard, hydrochlorothiazide increased Na^+^ excretion in NCC knockout mice (Leviel et al., 2010; Eladari and Chambrey, 2011). Furthermore, the increased Na^+^ excretion by hydrochlorothiazide appears mediated through inhibition of the Na^+^-driven chloride–bicarbonate exchanger (NDCBE; *SLC4A8e*; Ware et al., 2017; Nadal et al., 2018; Xu et al., 2018) because increased Na^+^ excretion, at least at 50 mg/kg i.p. hydrochlorothiazide, was absent in NCC/NDCBE double-knockout mice (Leviel et al., 2010; Eladari and Chambrey, 2011; Eladari and Hübner, 2011; Sinning et al., 2017).

It should also be considered that the hydrochlorothiazide dose that reduced arterial pressure in the absence of NCC (Alshahrani et al., 2017a) yields a plasma concentration in the range that inhibits NDCBE. That is, hydrochlorothiazide doses that increased Na^+^ excretion in NCC knockout mice, *i.p.* hydrochlorothiazide 4 and 50 mg/kg (Leviel et al., 2010; Eladari and Chambrey, 2011), correspond to plasma concentrations of at least 10 and 100 µM, respectively (Dose/Concentration of TZD). In fact, the hydrochlorothiazide concentration used in the metabolic studies in NCC/pendrin double-knockout mice, 40 mg/kg, would yield a plasma concentration of approximately 100 µM (Alshahrani et al., 2017a; Dose/Concentration of TZD). Further, 100 µM hydrochlorothiazide inhibited NDCBE in perfused renal cortical collecting ducts of mice (Leviel et al., 2010). In comparison, the IC_50_ for metolazone inhibition of Na^+^ uptake by NCC was 2 or 0.3 µM (depending upon the report; Sabath et al., 2004; Moreno et al., 2006). Also, essentially complete inhibition of NCC by metolazone occurred at 10 µM (Sabath et al., 2004; Moreno et al., 2006).

On the other hand, 10 mg/kg i.p. hydrochlorothiazide was selective for NCC as demonstrated in STE20/SPS1-related proline/alanine-rich kinase (SPAK)-dead knock-in mice (Glover and O’Shaughnessy, 2013). Specifically, in SPAK-dead knock-in mice, which demonstrated decreased NCC expression and phosphorylation leading to overall decreased NCC activity, hydrochlorothiazide saliuresis was absent (Rafiqi et al., 2010; Glover and O’Shaughnessy, 2013). Although NKCC2 expression and phosphorylation were also decreased in SPAK-dead knock-in mice (Rafiqi et al., 2010; Glover and O’Shaughnessy, 2013), saliuresis to the loop diuretic, furosemide, was not decreased (Rafiqi et al., 2010; Glover and O’Shaughnessy, 2013). However, whether NDCBE function is also decreased in SPAK-dead knock-in mice is not known (to our knowledge).

Although a lowered IC_50_ for TZD inhibition due to increased NDCBE expression following NCC knockout (Eladari and Chambrey, 2011) could occur, depending upon the presence of NDCBE spare transporters, a lowered IC_50_ does not appear to occur at least in Gitelman’s syndrome. In support of this conclusion is the decreased Na^+^ and Cl^−^ excretion with a single therapeutic dose of hydrochlorothiazide (*p.o.* 50 mg; Colussi et al., 1997; Peng et al., 2017; below).

While NDCBE-mediated TZD reduction of arterial pressure in NCC knockout mice subjected to Na^+^-restricted diet and NCC/pendrin double-knockout mice (Alshahrani et al., 2017a) may occur through hyponatremia, it should also be considered that hyponatremia may not accompany increased Na^+^ excretion. However, TZD-induced hyponatremia has been associated with decreased NCC (Channavajjhala et al., 2018). In fact, hyponatremia was observed in approximately one of seven TZD-treated patients (Glover and Clayton, 2012; Burst et al., 2017). Further, in patients with demonstrated hyponatremia treated with hydrochlorothiazide plus amiloride, challenge with 50 mg of hydrochlorothiazide, albeit combined with 5 mg of amiloride, caused hyponatremia (Friedman et al., 1989).

A major caveat, however, with respect to the potential functional role of NDCBE in Na^+^ handling and, therefore, possible high dose TZD-induced saliuresis and/or natriuresis, is the location of NDCBE within the kidney. Specifically, it was suggested that NDCBE was located in β-intercalated cells of the cortical collecting duct, consistent with saliuersis/natriuresis regulation (Ware et al., 2017; Nadal et al., 2018). In direct contrast, we were unable to locate NDCBE in cortical collecting duct β-intercalated cells (Xu et al., 2018). Moreover, NDCBE was restricted to the basolateral membrane of the medullary collecting duct (Xu et al., 2018), thereby questioning the potential role of NDCBE in Na+ handling.

#### Summary and Conclusions: NCC Knockout and NCC/Pendrin Double-Knockout Mice

TZD reduction of arterial pressure in NCC knockout mice subjected to Na^+^-restricted diet, but not NCC knockout mice with normal Na^+^ diet, and an even greater pressure reduction in NCC/pendrin double-knockout mice (Alshahrani et al., 2017a) are consistent with the dependency of TZD reduction of arterial pressure on decreased plasma volume *per se* and associated compensatory vasoconstriction (Diuresis and Plasma Volume; **Figure 2**). Thus, the considerable diuresis associated with less reduction of arterial pressure with NKCC2 inhibitors compared with TZD (Wilson and Freis, 1959; Anderson et al., 1971; Araoye et al., 1978; Holland et al., 1979; Diuresis and Plasma Volume) would reflect unopposed compensatory vasoconstriction with the NKCC2 inhibitors.

However, while TZD-reduced arterial pressure in NCC knockout mice with Na^+^-restricted diet and an even greater pressure reduction in NCC/pendrin double-knockout mice are suggestive of an extrarenal TZD target, an involvement of hyponatremia due to NDCBE inhibition cannot be completely eliminated because of the use of supra-therapeutic TZD dose (Alshahrani et al., 2017a). In fact, lack of TZD reduction of arterial pressure in Gitelman’s syndrome (below) at least indirectly supports the possibility of NDCBE inhibition in TZD reduction of arterial pressure. Overall, however, considering that NDCBE is apparently located on the basolateral membrane of medullary collecting duct (Xu et al., 2018) rather than in β-intercalated cells of the cortical collecting duct (Ware et al., 2017; Nadal et al., 2018), the function of NDCBE in response to hydrochlorothiazide needs to be revisited.

#### Gitelman’s Syndrome

Gitelman’s syndrome is an autosomal recessive malady attributed to dysfunctional NCC, largely due to missense mutations (Riveira-Munoz et al., 2007a; Riveira-Munoz et al., 2007b; Wang et al., 2015a). These mutations result in undetectable or reduced NCC amounts in urinary exosomes (Joo et al., 2007; Isobe et al., 2013; Yang et al., 2013). Consistent with NCC dysfunction in Gitelman’s syndrome is the lesser increase in urinary excretion of Na^+^ and Cl^−^ to hydrochlorothiazide (single, therapeutic dose of *p.o.* 50 mg; **Table 1k**; Dose/Concentration of TZD). In contrast, urinary excretion of Na^+^ and Cl^−^ increased in response to furosemide (loop diuretic; *i.v.* bolus infusion of 40 mg in Colussi et al., 1997 and *i.m.* 20 mg in Peng et al., 2016).

The phenotype associated with Gitelman’s syndrome includes hypomagnesemia, hypocalciuria, hypochloremia, and hypokalemic alkalosis (**Table 1I**; mimicked by NCC knockout mice; Schultheis et al., 1998; Alshahrani et al., 2017a; above). Vascular volume depletion is also associated with Gitelman’s syndrome, which results in elevated plasma renin levels/activity and aldosterone levels (Tsukamoto et al., 1995; Simon et al., 1996). Hypotension occurs in approximately 50% of patients with Gitelman’s syndrome (62% and 12% of patients and control subjects, respectively; Cruz et al., 2001). Based on measurements in neutrophils and monocytes from patients with Gitelman’s syndrome, the hypotension and absence of hypertension despite elevated plasma renin levels/activity and aldosterone levels were attributed to up-regulation/down-regulation of signaling pathways that would result in decreased vasoconstriction and basal vascular tone (Calò et al., 1998, Calò et al., 1999; Calò, 2006). These signaling pathways include decreased inositol trisphosphate release of intracellular Ca^2+^, decreased activation of protein kinase C, increased nitric oxide formation due to increased endothelial nitric oxide synthase expression, inhibition of oxidation signaling mechanisms (which mediate vasoconstriction to angiotensin II and other constrictors), decreased G-protein coupling through reduced α_q_ expression, decreased agonist activation of Rho kinase through down-regulation of RhoA/Rho kinase signaling, and increased heme-oxygenase-1 expression (Calò et al., 1998, Calò et al., 1999; Calò, 2006).

However, possibly inconsistent with the predicted decreased agonist constriction and/or basal vascular tone in patients with Gitelman’s syndrome (Calò et al., 1998, Calò et al., 1999; Calò, 2006) are 1) autonomic function was preserved in hypotensive, Gitelman’s syndrome patients (Sartori et al., 2007). Autonomic function was determined indirectly by reflex tests including the Valsalva maneuver, cold pressor, hand grip, and hyperventilation and by norepinephrine and arginine vasopressin plasma levels in response to the reflex tests (Sartori et al., 2007). And 2) basal blood flow in the forearm of two hypotensive patients with Gitelman’s syndrome did not differ from basal blood flow in normotensive and hypertensive subjects (Pickkers et al., 1998).

Also, possibly inconsistent with the potential extrarenal TZD target in NCC knockout mice subjected to Na^+^-restricted diet and in NCC/pendrin double-knockout mice (Alshahrani et al., 2017a), in two patients with Gitelman’s syndrome with 8 and 25 µg hydrochlorothiazide/min/dL forearm infusion (plasma hydrochlorothiazide concentrations of 2.7 and 11.0 µM, respectively), blood flow did not increase (Pickkers et al., 1998). Similarly, while 75 µg hydrochlorothiazide/min/dl infusion (38 µM of hydrochlorothiazide) increased forearm blood flow in Gitelman’s syndrome patients, the magnitude of increase was not different from the increased blood flow in normotensive and hypertensive subjects (Pickkers et al., 1998). The increased blood flow to 75 µg hydrochlorothiazide infusion likely resulted from non-specific effects to the approximately 20-fold greater than therapeutic plasma levels of hydrochlorothiazide (Pickkers et al., 1998; Dose/Concentration of TZD).

Additionally, based upon the acute hydrochlorothiazide reduction of arterial pressure in NCC knockout mice subjected to Na^+^-restricted diet and in NCC/pendrin double-knockout mice (Alshahrani et al., 2017a; above), TZD may have been predicted to reduce arterial pressure in patients with Gitelman’s syndrome. However, hydrochlorothiazide infusion into the forearm also did not reduce arterial pressure in the two Gitelman’s syndrome patients (as well as the normotensive and hypertensive subjects; Pickkers et al., 1998). The limited duration of hydrochlorothiazide infusion, i.e., 5 min, however, may have been insufficient to determine whether arterial pressure was actually reduced (Pickkers et al., 1998). Arterial pressure was also not reduced following diagnostic challenge with *p.o.* 50 mg of hydrochlorothiazide in Gitelman’s syndrome patients (L Chen and G Colussi, personal communications), with heart rate also remaining unchanged (G Colussi, personal communication). Lack of hydrochlorothiazide arterial pressure reduction in patients with Gitelman’s syndrome appeared independent of basal arterial pressure, as hydrochlorothiazide failed to reduce arterial pressure in Gitelman’s syndrome patients with low-normal mean arterial blood pressures, ranging at basal from 73 to 96 mmHg (*n* = 5; G Colussi, personal communication).

On the other hand, plasma volume depletion in patients with Gitelman’s syndrome may be insufficient to demonstrate TZD reduction of arterial pressure, consistent with the inability of TZD to reduce arterial pressure in NCC knockout mice with normal Na^+^ diet (Alshahrani et al., 2017a). However, significant plasma volume depletion likely occurs in Gitelman’s syndrome based on the elevated plasma renin levels/activity and aldosterone levels (Tsukamoto et al., 1995; Simon et al., 1996). Further, treatment of Gitelman’s syndrome, a condition associated with high Na^+^ intake, includes *ad libitum* dietary Na^+^ (Blanchard et al., 2017). Despite the increased Na^+^ intake, the intake apparently is insufficient to fully compensate for the decreased plasma volume (Diuresis and Plasma Volume) and, therefore the contribution of the decreased plasma volume to the hypotension.

#### Summary and Conclusions: Gitelman’s Syndrome

Compensation of contracted plasma volume in Gitelman’s syndrome appears to occur through elevated aldosterone and renin and possibly other vasoconstrictor pathways (Simon et al., 1996). Hypotension associated with Gitelman’s syndrome, along with lack of hypertension despite increased renin activity and aldosterone plasma levels (Simon et al., 1996), suggest both an incomplete compensation and a prevention of increased constriction due to reduced vascular contractility (Calò et al., 1998, Calò et al., 1999; Calò, 2006). However, unaltered autonomic nervous system regulation of arterial blood pressure and absence of changes in basal and TZD-mediated forearm blood flow in patients with Gitelman’s syndrome (Pickkers et al., 1998; Sartori et al., 2007) do not appear to support a mechanism whereby up-regulation or down-regulation of these non-selective vascular inhibitory and excitatory signaling pathways, respectively, underlies hypotension and inhibition of anticipated elevated arterial pressure due to apparent activation of the renin–angiotensin II–aldosterone pathway (Calò et al., 1998, Calò et al., 1999; Calò, 2006).

Thus, it is tempting to conclude that selective inhibition of the renin–angiotensin II–aldosterone pathway underlies the compromised vascular constriction. This conclusion appears consistent with lower plasma renin and urine aldosterone levels as predictors of greater TZD efficacy of arterial pressure reduction (Chapman et al., 2002; Diuresis and Plasma Volume). A further speculation is that alterations in blood electrolytes and pH associated with Gitelman’s syndrome, i.e., hypomagnesemia, hypocalciuria, hypochloremia, and hypokalemic alkalosis (**Table 1l**), underlie possible compromised renin–angiotensin II–aldosterone pathway. However, it appears that these conditions do not decrease the formation of angiotensin II (Tsukamoto et al., 1995).

The absence of TZD reduction of arterial pressure in Gitelman’s syndrome is not supportive of an extrarenal TZD target suggested by TZD reduction of arterial pressure in NCC knockout mice subjected to Na^+^-restricted diet and in NCC/pendrin double-knockout mice (Alshahrani et al., 2017a). However, this apparent lack of support could be explained if intravascular volume contraction is less in Gitelman’s syndrome than in knockout mice models (above).

### (4) Constrictor Pathways *In Vivo*

#### (a) Endogenous Activation

An additional assessment of contracted plasma volume in response to TZD is determination of compensatory constrictor pathways. Moreover, TZD may inhibit the compensatory vasoconstriction, eliciting reduced vascular tone and resulting in only partial compensation of the arterial pressure reduction. Additionally, these same constrictor pathways may be activated in some types of hypertension. Thus, determination of the effects of TZD on these constrictor pathways is fundamental to the identification of the TZD extrarenal target. The involvement of constrictor pathways can be assessed in part through determinations of endogenous levels of humoral and neurogenic constrictor factors and effects of TZD on constriction by these factors. Major compensatory constrictor pathways include the renin–angiotensin II–aldosterone and autonomic nervous systems. Overall, however, the relevance of plasma renin activity and angiotensin II plasma levels is unclear due to the importance of intrarenal versus systemic levels of these factors (Reudelhuber, 2013).

#### Renin–Angiotensin II–Aldosterone System

##### Responders and TZD-Sensitive Hypertension Models

A varied relationship exists between TZD-induced changes in renin activity/angiotensin II levels in plasma and reduction in arterial pressure (**Figure 1**). In hypertensive patients, chronic TZD-mediated increased renin activity/angiotensin II levels were maintained, partially maintained, or returned to levels not significantly different from pre-TZD levels (**Table 1m**).

However, conclusions that chronic TZD failed to elevate renin activity/angiotensin II levels are complicated by the underlying varied relationship between hypertension and plasma renin activity (Alderman et al., 2004; Atlas, 2007). An additional complication is the inclusion of a significant number of patients on additional antihypertensive medications, although acute TZD increased renin activity despite inclusion of multi-antihypertensive-treated patients (Bourgoignie et al., 1968). The detection of increased renin activity with acute but not chronic TZD may reflect a greater increase in the former (Bourgoignie et al., 1968). In the additional study that demonstrated a lack of increased renin activity/angiotensin II levels with chronic TZD, the patient population could be divided into those with and those without elevated plasma angiotensin II levels (Xiao et al., 2013). Arterial pressure was not reported, and, thus, possible association between TZD arterial pressure reduction and plasma angiotensin II levels remained unexamined (Xiao et al., 2013). Similar to elevated renin activity/angiotensin II levels in response to TZD, Na^+^-restricted diet also increased renin expression/angiotensin II levels in humans (Luft et al., 1991; Pimenta et al., 2009).

TZD-induced changes in aldosterone plasma levels generally correlated with increased renin activity/angiotensin II plasma levels (**Figure 1**). This correlation is based, however, upon a limited number of measurements of aldosterone plasma levels with and, for that matter, without renin activity/angiotensin II plasma levels in response to TZD. In fact, aldosterone levels tended to be elevated and were not significantly elevated when renin activity/angiotensin II levels were increased at 5 weeks and 3 months TZD treatment in humans, respectively (Maxwell and Gross, 1979; Lijnen et al., 1981). Further, renin activity/angiotensin II levels were at baseline at 2 months TZD treatment (Bourgoignie et al., 1968; Xiao et al., 2013). Similarly, aldosterone levels in the urine were not elevated following 3 days to 2 months of treatment with TZD (Gifford et al., 1961).

Enhanced TZD reduction of arterial pressure occurred with angiotensin receptor antagonists and angiotensin-converting enzyme inhibitor in human and spontaneously hypertensive rat (**Table 1**p), as well as with a mineralocorticoid receptor antagonist in human (Hollander et al., 1960).

##### Normotensive Humans and Animals

Renin activity/angiotensin II and aldosterone plasma levels in normotensive humans in response to TZD are largely unreported. Consistent with TZD-increased renin activity/angiotensin II levels, juxtaglomerular granularity increased following 9 weeks’ treatment with TZD in rat (Tobian et al., 1962). Na^+^-restricted diet also increased renin expression/angiotensin II levels in humans and NCC knockout mice and elevated Na^+^ diet decreased angiotensin II levels in humans (Genest et al., 1964; Alshahrani et al., 2017a).

Based upon the similar changes in plasma volume in responders and normotensive humans (Freis et al., 1960; Hollander et al., 1960; Diuresis and Plasma Volume), it is predicted that TZD increased renin activity/angiotensin II levels, as well as aldosterone levels, will be similar to those of responders (**Figure 1**). Na^+^-restricted diet increased aldosterone levels in humans (Genest et al., 1964).

##### Responders versus Nonresponders

TZD tended to increase renin activity/angiotensin II and aldosterone plasma levels to a greater magnitude in nonresponders than responders (van Brummelen et al., 1980; Freis et al., 1988; **Table 2**). It was suggested that the lack of arterial pressure reduction was due to compensatory angiotensin II vasoconstriction (van Brummelen et al., 1980; Freis et al., 1988). In contrast, TZD failure to increase renin activity and aldosterone levels in nonresponders was also observed (Svendsen et al., 1983), presumably due to underlying compensatory constrictor mechanisms.

#### Autonomic Nervous System

##### Responders and TZD-Sensitive Hypertension Models

Plasma levels of norepinephrine remained elevated with chronic TZD, consistent with increased sympathetic activity (Lake et al., 1979). Also consistent with the increased norepinephrine plasma levels (Lake et al., 1979), chronic TZD in hypertensive humans increased cardiac output/rate, although increased cardiac output/rate was also not observed (**Table 1n**). However, sympatholytic procedures and agents employed in the past to reduce arterial pressure, i.e., ganglionic blockers, reserpine, and sympathectomy, enhanced or did not prevent TZD arterial pressure reduction (**Table 1o**).

##### Normotensive Humans and Animals

As in some responders and TZD-sensitive hypertension models (**Table 1n**), heart rate was unaltered with chronic TZD in dogs (Jandhyala et al., 1972). Reserpine enhanced the arterial pressure reduction due to supra-therapeutic TZD dose in dogs, which would tend not to support the involvement of the sympathetic nervous system (Preziosi et al., 1959). In fact, TZD-lowered catecholamine levels in a number of tissues in dogs and mice suggest TZD-decreased sympathetic activity (Preziosi et al., 1961).

##### Responders versus Nonresponders

Similar responses to the Valsalva maneuver of TZD-treated responders and nonresponders do not support a role for autonomic nervous system activity and cardiac output in the differential reduction of arterial pressure (de Carvalho et al., 1977; **Table 2**). Resting cardiac output remained unchanged in responders and nonresponders (de Carvalho et al., 1977), presumably due to compensatory mechanisms. In partial contrast, while acute TZD decreased cardiac output in responders and nonresponders, with chronic TZD cardiac output returned to pre-TZD levels in responders but not in nonresponders (van Brummelen et al., 1980).

##### Summary and Conclusions: Endogenous Activation

Increased renin activity/angiotensin II and norepinephrine plasma levels in hypertensive humans and animals are consistent with a remaining component of decreased plasma volume in response to chronic TZD (**Figure 1**). Thus, TZD-reduced arterial pressure, while due to inhibition of compensatory angiotensin II and norepinephrine vasoconstriction as well as presumably other compensatory constrictor pathways, is also mitigated by further compensatory vasoconstriction (**Figure 2**). Additionally, increased renin activity/angiotensin II plasma levels by Na^+^-restricted diet suggest that similar constrictor pathways mediate the compensatory vasoconstriction due to plasma volume reduction in response to lowered Na^+^ and chronic TZD plasma volume decrease. Furthermore, the lesser magnitude of elevated renin activity/angiotensin II plasma levels in nonresponders (**Table 2**) is consistent with the lack of plasma volume decrease and resultant absence of arterial pressure reduction.

It should be noted, however, that increased renin activity/angiotensin II levels in response to chronic TZD were not consistently observed in hypertensive humans and animals (**Figure 1**). Furthermore, renin activity/angiotensin II levels were not consistently greater in responders than nonresponders (**Table 2**). Clearly, the limited number and contrasting findings of renin activity/angiotensin II levels, as well as aldosterone levels, warrant additional studies.

The enhancement or lack of prevention of TZD arterial pressure reduction by angiotensin receptor antagonists and angiotensin-converting enzyme inhibitor (**Table 1p**), mineralocorticoid receptor antagonists (Hollander et al., 1960), and ganglionic blockers, reserpine, and sympathectomy (**Table 1o**) appears inconsistent with TZD reduction of arterial pressure through inhibition of the renin–angiotensin–aldosterone and sympathetic nervous systems. Alternatively, TZD arterial pressure reduction would be enhanced by agents and procedures that disrupt the renin–angiotensin–aldosterone and sympathetic nervous system if TZD only partially inhibit activation of these systems.

The inability of TZD to reduce arterial pressure in neurogenic, i.e., sympathetic nervous system hypertension (**Table 1c**), also suggests that TZD inhibition of catecholamine-induced vasoconstriction is not involved in the reduction of arterial pressure. On the other hand, hypertension associated with high but not low renin activity/angiotensin II plasma levels is resistant to TZD reduction of arterial pressure (Chapman et al., 2002; **Table 1c**). The efficacy of TZD to reduce arterial pressure depending upon magnitude of renin–angiotensin II pathway activation may, by analogy, suggest that hypertension associated with high sympathetic nervous system activity is insensitive to arterial pressure reduction.

#### (b) Exogenous Agonist

TZD inhibition of the compensatory contracted plasma volume as well as constriction underlying certain types of hypertension may involve direct inhibition of vascular constriction. This assessment can be achieved in part through the effects of TZD on exogenous agonist pressor responses.

Numerous findings describe the effects of acute TZD on agonist-elevated and agonist-reduced vascular bed and mean arterial pressure (**Table 1q**). However, based upon the close correlation between acute TZD reduction of arterial pressure and diuresis, the extrarenal-mediated TZD reduction of arterial pressure is most likely absent during acute TZD (Diuresis and Plasma Volume). Thus, findings presented are limited to effects of chronic TZD on pressor responses (**Table 3**).

**Table 3:** Effect of Chronic Thiazide Diuretics on Agonist- and Sympathetic Nerve Stimulation-Elevated Arterial Pressure in Normotension and Hypertension.

Species	Hypertension	Duration Thiazide Diuretic	Pressure/Flow Measurement	Stimulant	Response^1^	Reference
Dog	No	7 days	MAP^2^	Norepinephrine	↑	Eckstein et al., 1962
	No	6–8 weeks	Hindlimb	Angiotensin II ATP Norepinephrine SNS^3^	− − ↓ ↓	Zsotér et al., 1970
	No	12 months	Hindlimb	Norepinephrine SNS	− −	Jandhyala et al., 1972
Human	Essential	8 weeks	MAP	Norepinephrine	−	Hollander et al., 1960
	No	14 days	Digital Brachial	Norepinephrine	−, ↑ −, ↑	Mendlowitz et al., 1960
	No	7 days	Forearm	Norepinephrine	−	Feisal et al., 1961
	Essential	6 days	MAP	Norepinephrine	↓	Aleksandrow et al.,1959
	Essential	14 days	Digital Brachial	Norepinephrine	↓	Mendlowitz et al., 1960
Rat	No	14 days	Hindlimb	Angiotensin II Epinephrine Norepinephrine	− − −	Aoki and Brody, 1969
	No	7 days	MAP	Norepinephrine	−	Neuvonen, 1971
	Renal^4^	14 days	Hindlimb	Angiotensin II SNS Tyramine	↓ ↓ −	Aoki and Brody, 1969
	Spontaneous	7 days	MAP	Norepinephrine	↓	Neuvonen, 1971
	Kidney/DOCA-salt^5^	10 days	MAP	Norepinephrine SNS Tyramine	− − −	Finch et al., 1977
	No	7 days	MAP	Angiotensin II Norepinephrine	↓ ↓	Zhu et al., 2005

^1^↑, ↓, − = increase, decrease, no change; ^2^MAP = mean arterial pressure; ^3^SNS = sympathetic nerve stimulation; ^4^non-responder; ^5^pithed

##### Responders and TZD-Sensitive Hypertension Models

In hypertensive patients treated with TZD, norepinephrine pressor responses remained unaltered (Hollander et al., 1960; **Table 3**). In contrast, TZD reduced the norepinephrine increased digital, brachial, and mean arterial pressure (Aleksandrow et al., 1959; Mendlowitz et al., 1960; **Table 3**) and increased forearm basal flow at supra-therapeutic dose (Pickkers et al., 1998). TZD reduction of mean arterial pressure was independent of hypovolemia because similar magnitudes of TZD reduction of mean arterial pressure occurred in hypovolemic and euvolemic patients (Mendlowitz et al., 1960). On the other hand, the magnitude of TZD reduction of the norepinephrine pressor response exceeded the magnitude of arterial pressure reduction (Mendlowitz et al., 1960).

Although not challenged with agonist, venoconstriction due to deep breathing and the Valsalva maneuver decreased in TZD-treated hypertensive patients (Ogilvie and Schlieper, 1970). These findings are consistent with TZD lowering of neurogenic activity (Ogilvie and Schlieper, 1970).

The norepinephrine pressor response in spontaneously hypertensive rats was also decreased by TZD (Neuvonen, 1971; **Table 3**). In contrast, TZD did not decrease pressor responses to norepinephrine, tyramine, and sympathetic nerve stimulation in 1 kidney/DOCA-salt hypertensive rats (Finch et al., 1977; **Table 3**). The differential TZD reduction of agonist pressor response depending upon the agonist (**Table 3**; Finch et al., 1977; Neuvonen, 1971) is consistent with the dependency of TZD reduction of hypertension upon the mechanism underlying the hypertension (Gong et al., 2012).

##### Normotensive Humans and Animals

In normotensive humans, TZD decreased norepinephrine-elevated digital and brachial pressure, although norepinephrine did not consistently elevate pressure (Mendlowitz et al., 1960). Similarly, norepinephrine decreased forearm blood flow, and TZD increased the flow in Responders and Nonresponders (i.e., reduced the pressure; Feisal et al., 1961; **Table 3**). The TZD-increased flow was attributed to increased cardiac output, possibly as the result of decreased cardioregulatory reflexes (Feisal et al., 1961).

In support of this suggestion, TZD treatment increased cardiac output and caused lesser decreased heart rate as compared to untreated dogs (Eckstein et al., 1962; **Table 3**). TZD also further increased norepinephrine elevated mean arterial pressure (Eckstein et al., 1962; **Table 3**). Thus, it was suggested that desensitization of carotid sinus and baroreceptor reflexes was responsible for the norepinephrine-induced increased heart rate and cardiac output with chronic TZD, resulting in further elevation of mean arterial pressure (Eckstein et al., 1962; **Table 3**). However, a role for decreased cardiac output in chronic TZD reduction of arterial pressure has been discounted (Central Vasomotor Centers).

In dog hindlimb, TZD did not reduce the elevated pressure due to norepinephrine, epinephrine, angiotensin II, and/or sympathetic nerve stimulation (Aoki and Brody, 1969; Jandhyala et al., 1972; **Table 3**). However, tonic, non-sympathetic, neurogenic tone was decreased by chronic TZD (Jandhyala et al., 1972; **Table 3**). In contrast, also in dog hindlimb, TZD reduced the elevated pressure due to norepinephrine and sympathetic nerve stimulation, but not to angiotensin II and ATP (Zsotér et al., 1970; **Table 3**). However, whether TZD actually reduced norepinephrine elevated mean arterial pressure in the dogs was not reported (Zsoter et al., 1907). TZD was without effect (Neuvonen, 1971) or reduced the norepinephrine, as well as the angiotensin II pressor response in rats (Zhu et al., 2005; **Table 3**). Thus, similar to the different efficacies of TZD to reduce arterial pressure depending upon the type of hypertension (**Table 1b and c**), there is varied TZD decrease in agonist pressor response.

##### Responders versus Nonresponders

TZD reduced elevated pressure to sympathetic nerve stimulation, angiotensin II, and norepinephrine, but not to tyramine, in the perfused hindlimb of renal hypertensive rats (Aoki and Brody, 1969; **Table 3**). The relevancy of the reduced pressor responses is unclear because TZD did not decrease mean arterial pressure in renal hypertensive rats (Aoki and Brody, 1969; **Table 3**).

##### Summary and Conclusions: Exogenous Agonist

Varied TZD reduction of norepinephrine-elevated arterial pressure, along with lack of correlation between these reductions and chronic TZD-reduced mean arterial pressure, casts doubt upon the involvement of possible decreased norepinephrine pressor response in arterial pressure reduction. Few, if any, studies determined TZD effects on pressor responses to agonists other than norepinephrine.

### (5) Vascular Contractility *In Vitro*

An additional assessment of TZD inhibition of increased tone due to plasma volume reduction and associated with hypertension is TZD inhibition in the isolated vasculature. The direct inhibition of increased tone can be determined by the effects of TZD a) *in vivo* on subsequently isolated vasculature and b) *in vitro* on agonist constriction.

#### (a) ***In Vivo*** TZD on Vascular Contractility Determined ***In Vitro***

##### Responders and TZD-Sensitive Hypertension Models

In femoral artery from hypertensive rats due to nitric oxide synthase inhibitor, a 7-week challenge with hydrochlorothiazide, which lowered the elevated arterial pressure, failed to increase acetylcholine-induced relaxation in isolated femoral artery (Sládková et al., 2007; **Table 4**).

**Table 4:** Effect of Thiazide Diuretics *In Vivo* on Vascular Constriction *In Vitro*.

Species	Preparation	Method	Hyper-tension	Duration Thiazide	Dose Thiazide^1^	Agonist	Thiazide Effect^2^	Reference
Dog	Mesenteric a.	Perfused	No	6 months 12 months	10 10	Ang II, NE N.S. Ang II, NE N.S.	− ↑ −	Clarke et al., 1972
Rabbit	Mesenteric a. Mesenteric v. Mesenteric v.	Isometric	No	3–4 weeks 6–10 weeks 6–10 weeks	∼5 ∼15 ∼15	NE NE NE Ang II, BaCl_2_, ACh Pap, ATP	− −^3^ ↓ − −^4^	Zsotér et al., 1970
Rat	Femoral a.	Isometric	L-NAME	7 weeks	40	ACh	−^4^ − −	Sládková et al., 2007

a., artery; ACh, acetylcholine; Ang II, angiotensin II; N.S., nerve stimulation; NE, norepinephrine; Pap, papaverine.;v., vein.

^1^mg/kg/day po; ^2^↑, ↓, − = increase, decrease, no change; ^3^tended to decrease; ^4^relaxation.

##### Normotensive Animals

In mesenteric arterial bed isolated from normotensive dogs, a 10-month challenge with hydrochlorothiazide failed to reduce norepinephrine-, angiotensin II-, and nerve stimulation-elevated perfusion pressure (Clarke et al., 1972; **Table 4**). In possible contrast, in rabbits challenged with hydrochlorothiazide for 6–10 weeks, constriction of mesenteric artery and vein to norepinephrine tended to decrease and decreased, respectively (Zsotér et al., 1970; **Table 4**). However, the significance of the reduced norepinephrine constriction is not clear because the vein is a capacitance vessel (Zsotér et al., 1970; **Table 4**). Moreover, norepinephrine constriction was not decreased following up to 4 weeks of challenge with hydrochlorothiazide (Zsotér et al., 1970; **Table 4**).

#### (b) ***In Vitro*** TZD on Vascular Contractility

Numerous studies investigated TZD inhibition of vascular contractility *in vitro* (**Tables 1r** and **5**). Moreover, a number of these studies, but not all, demonstrated TZD inhibition of constriction (**Tables 1s** and **5**). Proposed mechanisms underlying the inhibition of vascular contractility include carbonic anhydrase inhibition, increased nitric oxide release, BKCa^2+^ channel activation, decreased extracellular/intracellular Na^+^, decreased sensitization to intracellular Ca^2+^, RhoA and Rho kinase down-regulation, and inhibition of voltage-dependent Ca^2+^ channel activation (**Table 1s**).

However, i) supra-therapeutic TZD dose (Dose/Concentration of TZD) was required in a majority of studies to demonstrate inhibition of vasoconstriction (**Table 5**). Even though rabbits challenged with hydrochlorothiazide for 7 weeks with the supra-therapeutic dose of 40 mg/kg/day failed to increase acetylcholine relaxation of isolated femoral artery, while incubation of rabbit isolated femoral artery with 100 µM hydrochlorothiazide enhanced acetylcholine relaxation (Sládková et al., 2007; Vascular Contractility *In Vitro*, *In Vivo* TZD on Vascular Contractility Determined *In Vitro*; **Tables 4** and **5**). Indeed, the requirement of supra-therapeutic concentrations *in vitro* is similar to findings in humans of TZD-increased forearm blood flow, which required at least 38 µM of hydrochlorothiazide (Pickkers and Hughes, 1995; Pickkers et al., 1998), and in dogs, which required 9–18 times the usual therapeutic dose of chlorothiazide (Overbeck and Haddy, 1960). Concentration–response (inhibition of constriction) curves to TZD were not performed in a number of investigations (**Table 5**). Thus, TZD potency in these investigations remains to be established. ii) The magnitude of TZD inhibition of constriction depended significantly upon the TZD (Afsar et al., 2016). iii) Conclusions regarding mechanism underlying the inhibition apparently depended upon the contractile agent and/or vessel as observed, e.g., with the role of BKCa^2+^ channel inhibition (Calder et al., 1992, Calder et al., 1993, Calder et al., 1994; Colas et al., 2000a; **Table 5**). iv) *In vitro* effects may differ from *in vivo* effects. For example, the proposal that TZD decreased constriction through BKCa^2+^ channel inhibition based upon *in vitro* findings (Colas et al., 2000a; **Table 5**) is not supported by observations that *i.p*. paxilline, a BKCa^2+^ channel blocker, and a BKCa^2+^ channel/NCC/pendrin in triple-knockout mice did not prevent acute, hydrochlorothiazide reduction of arterial pressure (Alshahrani et al., 2017a). And v) vessels were not obtained from animals chronically treated with TZD (**Table 5**).

**Table 5:** Effect of Thiazide Diuretics *In Vitro* on Vascular Constriction^1^.

						Effect on Contractility^2,3^		
Species	Preparation	Method	Hypertension	Agonist	Inhibitor	TZD	Compared to TZD alone	Reference
Guinea pig	Mesenteric a.	Isometric	No	NE	none	↓	N/A	Calder et al., 1992
				NE	Glib	N/A	–	
				NE	Indo	N/A	–	
				NE	CTX	N/A	↓	
				NE	-Endo	N/A	–	
				KCl	none	–	NA	
				KCl + Ca^2+^	none	↓	N/A	Calder et al., 1993
				KCl + Ca^2+^	IBT	N/A	↓	
				NE	none	↓	NA	Calder et al., 1994
				NE	Apamin	N/A	–	
				NE	Glib	N/A	–	
				NE	Phen	N/A	–	
				NE	CTX	N/A	↓	
				NE	none	↓	N/A	Pickkers and Hughes, 1995
				NE	CTX	N/A	↓	
				NE + Ca^2+^	KCl	N/A	↓	
				NE	none	↓^4^	N/A	Pickkers et al., 1999
				NE	CTX	N/A	↓	
Human	Internal mammary a.	Isometric	Yes^5^	NE	none	↓	N/A	Afsar et al., 2016
				NE	Glib	N/A	–	
				NE	CTX	N/A	↓	
				KCl	none	–	N/A	
	Mesenteric a.	Isometric	No	NE	none	↓	N/A	Colas et al., 2001
			Essential^6^	NE	none	↓	N/A	
	Sub- cutaneous a.	Isometric	No	NE	none	↓	N/A	Calder et al., 1992
				NE	Glib	N/A	–	
				NE	Indo	N/A	–	
				NE	CTX	N/A	↓	
Mouse	Aorta	Isometric	No^7^	Phe	none	–	N/A	Alshahrani et al., 2017a
	Mesenteric arteriole	Perfused	No^7^	Phe	none	–	N/A	Rapoport et al., 2019
Rabbit	Aorta	Isometric	No	NE	none	–		Daniel and Nash, 1965
Rat	Caudal a.	Perfused	No	Ang II	none	–	N/A	Nicholas, 1970
				N.S. + Ang II	none	–	N/A	
				N.S.	none	–	N/A	
				None	none	–	N/A	
	Aorta	Isometric	No	Phen	none	↓^8^	N/A	Abrahams et al., 1996; Abrahams et al., 1998
			No	Phe	-Endo	N/A	↓^8^	
			Spontaneous	KCl + Ca^2+^	none	↓	N/A	Colas et al., 2000a
				KCl + Ca^2+^	L-NNA	–	↓	
				KCl + Ca^2+^	-Endo	N/A	↓	
				KCl + Ca^2+^	CTX	N/A	–	
			No	NE	none	–	N/A	Colas et al., 2000b
				AVP	none	–	N/A	
			Spontaneous	NE	none	↓	N/A	
				NE	-Endo	N/A	↓	
				AVP	none	–	N/A	
				NE	-Endo	N/A	↓	
			No	NE	none	–	N/A	Colas et al., 2000c
				NE	none	N/A	↓	
			Spontaneous	NE	none	↓	N/A	
				NE	-Endo	N/A	↓	
				NE	L-NNA	–	N/A	
				NE	Indo	N/A	↓	
				KCl + Ca^2+^	none	↓	–	
			No	NE	none	↓	N/A	Zhu et al., 2005
				Ang II	none	↓	N/A	
				Ang II	L-NAME	N/A	–	
				Ang II	-Endo	N/A	–	
				KCl	none	–	N/A	
				BaCl_2_	none	–	N/A	
	Femoral a.	Isometric	No	ACh^9^	none	↑	N/A	Sládková et al., 2007
			L-NAME	ACh^9^	none	– ^10^	N/A	
	Portal v.	Isometric	No	KCl + Ca^2+^	none	↓	N/A	Mironneau et al., 1981
			No	Spontaneous	none	–	N/A	Abrahams et al., 1996
	Pulmonary a.	Isometric	No	Phe	none	↓^8^	N/A	Abrahams et al., 1996

^1^Adapted and expanded from Rapoport et al. (2019).

^2^Constriction unless relaxation noted.

^3^↑, ↓, − = increase, decrease, no change constriction/relaxation.

^4^Substantial and minimal inhibition by hydrochlorothiazide and bendroflumethazide, respectively.

^5^Hypertension, myocardial infarction, hyperlipidemia, treated with several pharmacologic agents.

^6^Treated with angiotensin II receptor antagonist, angiotensin converting enzyme inhibitor, hydrochlorothiazide.

^7^NCC knockout plus Na^+^ restricted diet.

^8^Inhibition requires plasma (Abrahams et al., 1996) or albumin (Abrahams et al., 1998).

^9^Relaxation.

^10^Tended to increase.

a., artery, ACh, acetylcholine; Ang II, angiotensin II; AVP, arginine vasopressin; CTX, charybdotoxin; Endo, endothelium; Glib, glibenclamide; IBT, iberiotoxin; Indo, indomethacin; L-NAME, N^G^-nitro-L-arginine methylester; L-NNA, N_ω_-nitro-L-arginine; NE, norepinephrine; N.S., nerve stimulation; Pap, papaverine; Phenc, phencyclidine; Phe, phenylephrine; TZD, thiazide diuretic; v., vein.

Generally consistent with the dependency of TZD reduction of arterial pressure on altered regulatory protein expression is the minimal and greatly reduced arterial pressure with acute infusion and with 3-day TZD treatment, respectively, in hypertensive, angiotensin II-salt rats (Ballew and Fink, 2001). Additionally, TZD inhibition of vasoconstriction was attributed to decreased expression of proteins that sensitize agonist constriction, RhoA and Rho kinase (Zhu et al., 2005; **Table 4**). This conclusion was based in large part on decreased expression of these proteins with TZD challenge of cultured vascular smooth muscle cells (Zhu et al., 2005). Consistent with these findings is the more recent demonstration that chronic hydrochlorothiazide reduced arterial pressure in the hypertensive 1 kidney/DOCA-salt rat and decreased the associated elevated Rho-kinase (as well as gene expression of markers of remodeling) in the aorta (Araos et al., 2016).

On the other hand, decreased Rho-kinase would be expected to inhibit and enhance agonist constriction and relaxation, respectively (Nakamura et al., 2003). However, 1) chronic TZD failed to consistently inhibit constriction and relaxation was not enhanced (Constrictor Pathways *In Vivo*, Exogenous Agonist; Vascular Contractility *In Vitro*, *In Vivo* TZD on Vascular Contractility Determined *In Vitro*; **Tables 3** and **4**); 2) while Rho-kinase inhibition reduces arterial pressure in rats with normal arterial pressure (Dhaliwal et al., 2007), chronic (and acute) TZD do not reduce arterial pressure in rat and other animals and in human (Introduction; Diuresis and Plasma Volume; **Table 1j** and d, **Figure 1**; unreported in Araos et al. (2016) is whether hydrochlorothiazide decreased mean arterial pressure in sham operated rats); 3) although hydrochlorothiazide *in vitro* at clinically relevant concentrations decreased Rho-kinase expression and agonist constriction, based upon the varied inhibitory effects of TZD on vasoconstriction *in vitro* as well as the use in many studies of supra-clinical TZD concentrations (Vascular Contractility *In Vitro*, *In Vitro* TZD on Vascular Contractility; **Tables 1r, s**, and **5**), it is should be considered that inhibition of Rho-kinase activity by TZD is indirect and requires chronic exposure (Araos et al., 2016). In contrast, the Rho-kinase inhibitor, fasudil (Y27632), causes direct inhibition (Nakamura et al., 2003) and 4) intrarenal rather than systemic angiotensin II may be the important modulator of vascular contractility (Reudelhuber, 2013; Constrictor Pathways *In Vivo*, Endogenous Activation). Thus, the possible role of decreased Rho-kinase activity in chronic TZD reduction of arterial pressure remains unclear.

##### Summary and Conclusions: Vascular Contractility In Vitro

Lack of inhibition of agonist-induced constriction in vessels isolated from animals chronically challenged with TZD and in NCC knockout mice subject to Na^+^-restricted diet and NCC/pendrin double-knockout mice, despite TZD reduction of arterial pressure (Clarke et al., 1972; Sládková et al., 2007; Rapoport et al., 2019; **Table 5**), suggests that the condition(s) allowing TZD to reduce arterial pressure is absent *in vitro*. Thus, these findings (Clarke et al., 1972; Sládková et al., 2007; Alshahrani et al., 2017a; Rapoport et al., 2019; **Table 5**) do not support proposed mechanisms of TZD inhibition of vasoconstriction based upon *in vitro* challenge with TZD (Vascular Contractility *In Vitro*; **Table 5**). Indeed, the presence of the TZD target *in vivo*, but not *in vitro*, is consistent with reversal of TZD chronic reduction of arterial pressure with plasma volume repletion (Diuresis and Plasma Volume).

TZD-induced decreased vasoconstriction could occur through inhibition/activation of smooth muscle signaling pathways or requires up-regulation/down-regulation of these pathways. However, it is unlikely that expression of a potential TZD target at least within the vasculature is up-regulated/down-regulated because i) arteries isolated from animals chronically treated with TZD failed to demonstrate decreased constriction and/or increased relaxation (Zsotér et al., 1970; Clarke et al., 1972; Sládková et al., 2007; **Table 3**); ii) agonist pressor responses were not decreased with chronic TZD in a number of animal and human investigations (**Table 3**); iii) differences in phenylephrine constriction were not detected (independent of structural change) in vessels isolated from wild type and both NCC/pendrin double knockout and NCC knockout plus Na^+^ diet-restricted mice (Alshahrani et al., 2017a; Alshahrani et al., 2017b; Rapoport et al., 2019; **Table 5**); iv) hydrochlorothiazide, at the supra-therapeutic dose of 20 mg/kg, reduced arterial pressure within 1 h (shortest time period examined in unanesthetized mice) in NCC knockout mice subjected to Na^+^-restricted diet and NCC/pendrin knockout mice (Alshahrani et al., 2017a). It is unlikely that 1 h is sufficient time to significantly alter protein expression. However, a caveat to this conclusion is that hydrochlorothiazide at this dose may reduce arterial pressure through hyponatremia, rather than an extrarenal target (Alshahrani et al., 2017a; [Na^+^/Cl^–^ Contransporter (NCC; SLC12A3)]); and v) chronic TZD *in vivo* with and without additional TZD challenge of vessels isolated from these animals did not inhibit agonist constriction (Zsotér et al., 1970; Clarke et al., 1972; Sládková et al., 2007; **Table 4**).

An array of mechanisms has been proposed for TZD direct inhibitory effects on vasoconstriction. However, the relevancy of these mechanisms is limited by the procedures adopted for these determinations (“b” above; *In Vitro* TZD on Vascular Contractility). Furthermore, with respect to overall considerations vis-à-vis measurements of vascular contractility—i.e., 1) the use of resistance type vessels rather than conduit vessels 2) the vessels should be derived from vascular beds associated with resistance, and 3) a relatively small change in contractility, which may or may not be detected depending upon measurement sensitivity, underlies a large change in blood flow, i.e., blood flow is proportional to the fourth power of the radius (Poiseulle’s law)—these considerations appear largely satisfied through recent video-imaging measurements of changes in diameter in mesenteric arterioles, i.e., resistance vessels (Rapoport et al., 2019). Moreover, the mesenteric vascular bed is relevant to determinations of peripheral vascular resistance and, thus, arterial blood pressure regulation (Heyndrickx et al., 1976; Christensen and Mulvany, 1993).

### (6) Central Vasomotor Centers

In addition to peripheral actions of TZD, potential central effects of TZD need to be considered. The relevancy of central effects of TZD is unlikely because central access of TZD following peripheral administration is limited, as demonstrated in patients with various neurological diseases (Sigaroudi et al., 2018).

#### Responders and TZD-Sensitive Hypertension Models

TZD injection into the hypothalamus reduced arterial pressure in spontaneously hypertensive rats (Bergmann and Altshuler, 1996). However, in hypertensive patients, TZD was neutral with respect to central systolic pressure (Morgan et al., 2004; Mackenzie et al., 2009; Kwon et al., 2013; Manisty and Hughes, 2013).

Direct TZD application to the carotid artery sinus in the presence and absence of norepinephrine and epinephrine failed to reduce arterial pressure in dogs with acute hypertension due to vagi-aortic nerve denervation (Preziosi et al., 1959; Preziosi et al., 1961). Concomitant with the findings related to baroreceptor reflexes (Preziosi et al., 1959; Preziosi et al., 1961), while TZD increased the gain of renal nerve activity and heart rate in spontaneously hypertensive rats, the increase was non-selective in that similar findings were observed with several classes of anti-hypertensive agents (Kumagai et al., 1996).

#### Normotensive Animals

TZD did not lower arterial pressure when infused into the carotid artery in a dog preparation with ligated internal and external carotid arteries with and without carotid sinus denervation (Preziosi et al., 1961). Consistent with these findings TZD were without effect on isolated guinea pig and rabbit heart (Preziosi et al., 1959).

#### Summary and Conclusions: Central Vasomotor Centers

It is unlikely that TZD act centrally at vasomotor centers to decrease peripheral vascular resistance. Further, the relevance of determinations of TZD effects in normotensive animals is unclear because TZD do not lower arterial pressure in normotensive humans and animals (Diuresis and Plasma Volume).

### (7) Other Signaling Pathways Based Upon Genetic Analysis

#### Hypertension Responders and Nonresponders

In terms of baseline protein expression in responders and nonresponders, responders had greater mRNA baseline levels of a number of proteins including vasodilator-stimulated phosphoprotein (VASP) and metabolites of the sphingolipid metabolic pathway (Turner et al., 2013; Shahin et al., 2017; Sá et al., 2018). It was suggested that increased baseline VASP expression and the sphingolipid metabolic pathway were responsible for TZD efficacy of arterial blood pressure reduction (Shahin et al., 2017).

#### Summary and Conclusions: Other Signaling Pathways Based Upon Genetic Analysis

The involvement of these pathways in TZD reduction of arterial pressure warrants additional investigation. Indeed, it remains unclear whereby, e.g., increased sphingolipid metabolism would result in greater efficacy of TZD reduction of arterial pressure (Shahin et al., 2017).

### (8) Guiding Parameters

Suggested required parameters for relevant investigations into extrarenal TZD target identification include the following:

(a) Therapeutic TZD dose/concentration: *In vivo* doses should be in the therapeutic range (Dose/Concentration of TZD). Indeed, higher TZD doses elicit several effects not relevant to TZD mechanism of action at therapeutic dose (Na+/Cl^−^Contransporter [NCC; *SLC12A3*]). *In vitro* concentrations should reflect TZD plasma levels (Dose/Concentration of TZD and Vascular Contractility *In Vitro*).
(b) Chronic TZD: The extrarenal TZD target is exposed/present with chronic TZD (Diuresis and Plasma Volume and Vascular Contractility *In Vitro*). Thus, the relevancy of acute TZD challenge *in vivo* and *in vitro* is unclear.
(c) TZD effect on normal arterial pressure: TZD should not reduce arterial pressure in normotensive animals. This stipulation is derived from the lack of effect of TZD on arterial pressure in normotensive humans and animals (Diuresis and Plasma Volume). Thus, the relevancy of acute TZD challenge on vascular reactivity in normotensive animals and in vascular tissue from normotensive animals is unclear (Constrictor Pathways *In Vivo;* Vascular Contractility *In Vitro*).
(d) TZD and hypertension: TZD should reduce arterial pressure in hypertensive models (“responders” in humans; Diuresis and Plasma Volume). Moreover, due to the dependency of TZD reduction of arterial pressure on underlying hypertension, findings should be derived from hypertensive models and from tissues obtained from these models (Diuresis and Plasma Volume; Vascular Contractility *In Vitro*).
(e) TZD and NCC: The acute effects of TZD should be mediated through NCC inhibition ([Na^+^/Cl^−^ Cotransporter (NCC; *SLC12A3*)]).
(f) Other diuretics: The TZD target should not be utilized by non-TZD, e.g., NKCC2 inhibitors (loop diuretics; Diuresis and Plasma Volume).
(g) TZD-insensitive hypertension (“nonresponders” in humans): The TZD target should not be exposed/present in hypertensive models insensitive to TZD arterial pressure reduction (Diuresis and Plasma Volume, [Na^+^/Cl^−^ Cotransporter (NCC; *SLC12A3*)]; Constrictor Pathways In Vivo).

### (9) Perspective

#### (a) TZD Target Site

It is unlikely that TZD selectively inhibit the presumably different TZD targets associated with each of the multiple types of hypertension sensitive to reduction by TZD (**Table 1c**; Introduction). For example, hypertension varies with respect to the magnitudes of sympathetic nerve activity and vascular contractile sensitivity (**Table 1t**). Thus, as illustrated in the working model (**Figure 2**), TZD reduction of arterial pressure is unrelated to direct inhibition of a specific constrictor pathway but rather through a common effect that inhibits constriction to multiple pathways. These multiple pathways underlie constriction (1) to TZD-decreased plasma volume (consistent with earlier proposals of Tapia et al., 1957; Freis et al., 1958; Dustan et al., 1959; Tarazi et al., 1970) and (2) responsible for the numerous TZD-sensitive types of hypertension (**Table 1c**; Introduction). Alternatively, a particular pathway sensitive to TZD inhibition, e.g., renin–angiotensin II, could underlie the multiple types of hypertension associated with responders.

In any case, the above proposal suggests that TZD reduction of arterial pressure is mediated by inhibition of underlying compensatory vasoconstrictor pathways (**Figure 2**). Indeed, removal of the compensatory constriction, through repletion of plasma volume by infusion with dextran plus saline/glucose or elevated dietary Na^+^, reversed TZD chronic reduction of arterial pressure (Freis et al., 1958; Wilkins et al., 1958; Wilson and Freis, 1959; Winer, 1961). Also consistent with a common TZD target is enhancement of agonist constriction with myogenic tone (Faber and Meininger, 1990).

It should also be noted that, despite the potential attractiveness of the suggestion that NCC serves as the extrarenal target underlying TZD chronic arterial pressure reduction based upon the lower arterial pressure in Gitelman’s syndrome (Calò, 2006), this suggestion is inconsistent with restriction of NCC expression to the kidney, i.e., as evidenced by lack of NCC expression and binding in the aorta, as well as the adrenal gland, brain, heart, intestine (large and small), liver, lung, pancreas, placenta, salivary gland, skeletal muscle, spleen, and testis in humans and/or rats (Beaumont et al., 1988; Hebert and Gamba, 1995; Mastroianni et al., 1996).

#### (b) TZD Arterial Pressure Reduction

As illustrated in the working model of **Figures 2A–D**:

(**A**) Normotensive subjects/animals: The proposed dependency of TZD arterial pressure reduction on inhibition of compensatory vasoconstrictor pathways (Guiding Parameters (a) Therapeutic TZD dose/concentration) suggests that the absence of TZD reduction of arterial pressure in normotensive subjects and animals (Diuresis and Plasma Volume) is due to complete mitigation by compensatory vasoconstriction of the TZD inhibition of constriction (**Figure 2A**).

(**B**) Decreased plasma volume: In contrast to the lack of TZD reduction of arterial pressure in normotensive subjects/animals, TZD reduced arterial pressure following enhanced plasma volume depletion, as observed in NCC knockout mice subjected to Na^+^-restricted diet and NCC/pendrin knockout mice (Alshahrani et al., 2017a). Thus, even greater compensatory vasoconstriction is required to limit the decrease in arterial pressure (Alshahrani et al., 2017a). However, a further decrease in plasma volume due to TZD cannot be fully compensated for by the already enhanced magnitude of compensatory vasoconstriction. The lack of complete mitigation of the plasma volume reduction, therefore, fails to fully compensate for TZD inhibition of vasoconstriction (**Figure 2B**). A caveat to this explanation is the possible mediation of TZD reduction of arterial pressure in NCC knockout mice subjected to Na^+^-restricted diet and NCC/pendrin knockout mice (Alshahrani et al., 2017a) through NDCBE inhibition ([Na^+^/Cl^−^ Contransporter (NCC; *SLC12A3*)]).

(**C**) TZD-sensitive hypertension: Similar to TZD reduction in arterial pressure following enhanced plasma volume depletion (Alshahrani et al., 2017a), in TZD-sensitive hypertension, the magnitude of constriction due to hypertension plus decreased plasma volume in response to TZD cannot fully mitigate TZD inhibition of constriction (**Figure 2C**). Thus, TZD reduces the elevated arterial pressure (**Figure 2C**). This proposal further suggests, therefore, that reduction of arterial pressure in hypertension requires an underlying component of reduced plasma volume. In this regard, this suggestion is consistent with the reversal of the TZD lowered arterial pressure with plasma volume repletion (Diuresis and Plasma Volume). It should also be considered, however, that a particular pathway sensitive to TZD inhibition, e.g., renin–angiotensin II, could underlie the multiple types of hypertension associated with responders (Guiding Parameters (a) Therapeutic TZD dose/concentration).

(**D**) TZD-insensitive hypertension: Considering the suggested requirement for an underlying component of reduced plasma volume to lower elevated arterial pressure (**C** above), the inability to decrease arterial pressure in some types of hypertension may be due to prevention of contracted plasma volume (**Figure 2D**). The lack of contracted plasma volume is consistent with the greater TZD-increased diuresis, plasma volume reduction, and body weight loss in responders than nonresponders and the inability of TZD to decrease plasma volume in nonresponders (de Carvalho et al., 1977; Svendsen et al., 1983; Freis et al., 1988; **Table 2**; Diuresis and Plasma Volume). Alternatively, the constrictor pathways underlying TZD-insensitive hypertension may overcome the TZD inhibition of constriction (**Figure 2D**).

Clearly, the identity of the extrarenal site of TZD inhibition of vascular tone remains for further investigation. Moreover, this site is presumably active in Gitelman’s syndrome. TZD-insensitive hypertensive models should also assist in this identification through delineation of factors in common with, and different from, TZD-sensitive hypertensive models and unique to particular models. Additionally, several genetic signals associated with sensitivity to TZD reduction of arterial pressure have been identified (Melander et al., 2013; Shahin and Johnson, 2016; Sá et al., 2018). Whether these or other signals underlie TZD inhibition of vasoconstriction remains for further investigation. Identification of the TZD extrarenal target will provide the basis for conversion of nonresponder to responder and the development of novel antihypertensive therapies acting through this site.

## Author Contributions

RR and MS conceived, RR wrote, and MS reviewed the article.

## Funding

This study was supported by a Merit Review award from the Department of Veterans Affairs (5I01BX001000), funds from the Center on Genetics of Transport and Epithelial Biology at the University of Cincinnati, and grants from the Dialysis Clinic, Inc. and US Renal Care (MS).

## Conflict of Interest Statement

The authors declare that the research was conducted in the absence of any commercial or financial relationships that could be construed as a potential conflict of interest.
